# NLSS3 Impairs SHM1 Autophagic Degradation to Regulate Leaf Morphology and Salt Tolerance in Rice

**DOI:** 10.1002/advs.75700

**Published:** 2026-06-03

**Authors:** Xiong Liu, Yulu Yang, Zhiqi Hao, Jing Xu, Huibo Zhao, Qiang Zhang, Deyong Ren, Xia Li, Guojun Dong, Lan Shen, Li Zhu, Jiang Hu, Zhenyu Gao, Qing Li, Qian Qian, Guangheng Zhang

**Affiliations:** ^1^ State Key Laboratory of Rice Biology and Breeding China National Rice Research Institute Hangzhou China; ^2^ Academician Workstation National Nanfan Research Institute (Sanya) Chinese Academy of Agricultural Sciences Sanya China; ^3^ State Key Laboratory of Tree Genetic and Breeding Zhejiang Key Laboratory of Forest Genetics and Breeding Research Institute of Subtropical Forestry Chinese Academy of Forestry Hangzhou China; ^4^ Wild Rice Germplasm Resources Innovation Team Yazhouwan National Laboratory Sanya China

**Keywords:** autophagic degradation, EXO70 protein, leaf morphogenesis, pleiotropic regulation, rice, salinity tolerance

## Abstract

The inherent trade‐off between optimal plant architecture and robust stress resilience represents a fundamental challenge in the breeding of high‐yielding rice cultivars. Overcoming this antagonistic relationship requires identifying master regulatory genes that can coordinately modulate both growth and stress response pathways. In this study, *NLSS3* (*Narrow Leaf and Salt Sensitive 3*) was identified as a pleiotropic regulator governing both leaf morphogenesis and salinity tolerance in rice. NLSS3 physically interacts with SHM1 (serine hydroxymethyltransferase), protecting it from autophagy‐mediated degradation and thereby enhancing its protein stability. The loss of NLSS3 function causes SHM1 depletion, resulting in serine deficiency and heightened salt sensitivity. Strikingly, *SHM1* overexpression not only restored leaf width in the *nlss3* mutant but also significantly increased grain yield and salt tolerance in wild‐type plants. Moreover, the superior *NLSS3* haplotype Hap1, which is predominant in *japonica* accessions, shows high expression and enhanced salinity tolerance. These findings establish NLSS3 as a central “autophagy guardian” that integrates plant development and environmental adaptation through the regulation of protein homeostasis. This work positions *NLSS3* as a promising target for precision breeding to engineer high‐yielding and stress‐resilient rice varieties.

## Graphical abstract

In rice, NLSS3 binds and shields SHM1 from autophagic degradation to maintain serine homeostasis. The A132P mutation in *nlss3* disrupts this, triggering SHM1 loss, metabolic dysfunction, K^+^ imbalance, and impaired ROS scavenging, which collectively drive narrow leaves and salt sensitivity. Superior haplotypes of *NLSS3* are associated with improved salt tolerance without compromising yield.

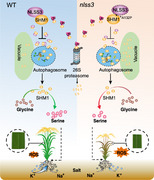

## 1 Introduction

Rice (*Oryza sativa* L.) stands among the most vital staple crops globally, serving as a primary food source for nearly half of the global population [1]. Plant architecture is a central determinant of rice morphological performance, encompassing agronomically critical traits such as tillering pattern, leaf morphology, panicle architecture, and root system configuration. While considerable efforts have been directed toward optimizing plant architecture to enhance yield potential, a persistent trade‐off between growth vigor and stress resilience often constrains simultaneous improvement. This phenotypic dilemma may be mitigated through the functional dissection of master regulatory genes that coordinate developmental and stress‐responsive pathways [2, 3]. The *SD1* (*SEMIDWARF1*) gene, encoding an enzyme in gibberellin biosynthesis, exemplifies such dual functionality. The semi‐dwarfism and improved lodging resistance conferred by the *sd1* allele are major contributors to the success of the green revolution in rice [4]. Paradoxically, in deepwater rice ecotypes, elevated *SD1* expression promotes internode elongation, facilitating submergence escape [5]. More recently, *SD1* has been implicated in abiotic stress adaptation, with precise modulation of its expression shown to concurrently enhance both yield and salt‐alkali tolerance, highlighting its pleiotropic regulatory potential [6]. Similarly, *IPA1 (IDEAL PLANT ARCHITECTURE1)*, another pivotal green revolution gene, boosts grain yield by directly binding the promoters of *DENSE PANICLE 1 (DEP1)* and *TEOSINTE BRANCHED 1 (TB1)* to regulate tillering and panicle branching [7, 8, 9], As a key transcription factor, IPA1 also integrates stress signaling by targeting *WRKY45* and *stress‐responsive NAC 1 (SNAC1)*, thereby modulating responses to both biotic and abiotic stress [10, 11, 12, 13]. Functional characterization of IPA1 has thus enabled a balanced optimization of agronomic performance and environmental resilience, offering a strategy to improve multiple agronomic traits simultaneously [14, 15].

Leaf morphology, a key component of plant architecture, serves as the main organ for photosynthesis and directly influences rice yield. The developmental regulation of leaf shape, particularly in relation to stress adaptation, represents a critical nexus for yield enhancement under adverse conditions. Emerging genetic studies have begun to unravel the molecular underpinnings of this trade‐off. *Photo‐sensitive leaf rolling 1 (PSL1)*, encoding a polygalacturonase, modulates pectin dynamics in the cell wall; its loss‐of‐function leads to pronounced leaf rolling under stress, which reduces water loss and significantly improves drought tolerance [16]. *Semi‐rolled leaf 10 (SRL10)*, which encodes a double‐stranded RNA‐binding protein, not only governs miRNA biogenesis to regulate leaf curvature but also physically interacts with CATB to stabilize catalase activity under heat stress, thereby reinforcing thermotolerance [17]. Additionally, DROUGHT AND SALT TOLERANCE (DST) directly regulates *Narrow leaf 1 (NAL1)* expression to control leaf width and modulates *OsNR1.2* under drought, linking leaf development with drought resilience [18, 19].

The EXO70 protein, a core subunit of the exocyst complex, plays a conserved role in vesicle tethering and exocytosis across eukaryotes [20, 21, 22]. The EXO70 gene family has expanded and diversified substantially in plants, with 23 members in *Arabidopsis thaliana* and 47 in rice, indicating significant functional divergence between these model species [23, 24]. Notably, emerging evidence points to a direct role for the exocyst complex and specific EXO70 isoforms in autophagy regulation [25]. AtEXO70B1 co‐localizes with ATG8f‐marked autophagosomes and facilitates their maturation [26], whereas AtEXO70D proteins function as selective autophagic receptors mediating the degradation of ARR‐type proteins [27]. In rice, only a subset of EXO70 genes have been functionally defined, including *OsEXO70F2/B1* and *OsEXO70H3*, which function in defense against pathogens and insects, respectively [28, 29, 30], and *OsEXO70E1/Short Root 1 (SR1)*, which regulates root development [31]. Despite these insights, the broader functional landscape of the EXO70 family in rice, particularly in leaf morphogenesis, autophagy, and abiotic stress responses, remains largely unexplored.

Serine hydroxymethyltransferase (SHMT) is a pivotal enzyme in photorespiration, catalyzing the conversion of glycine to serine in concert with glycine decarboxylase (GDC). In Arabidopsis, SHM1 deficiency results in a marked increase in the glycine /serine ratio [32], whereas its heterologous overexpression in *E.coli* elevates glycine and serine accumulation [33]. In Arabidopsis, *shm1* mutants exhibit hypersensitivity to salt stress, and the FERONIA (FER) receptor‐like kinase phosphorylates SHM1 to stabilize the protein, thereby modulating salt tolerance [34, 35, 36]. Microtubule‐associated C4HC3‐type E3 Ligase (MEL) promotes ubiquitin‐mediated degradation of SHMT1/SHM1 to enhance resistance to rice blast and bacterial blight [37]. Although OsSHM1 has been associated with photorespiratory flux and cold stress responses [38, 39], its involvement in plant architectural regulation or salt stress adaptation remains uncharacterized, and the underlying molecular mechanisms are yet to be elucidated.

Here, we report the identification of *Narrow Leaf and Salt sensitive 3* (*NLSS3*), a previously uncharacterized EXO70 family member that functions as a critical regulator of leaf development and salt tolerance in rice. We demonstrate that NLSS3 physically interacts with OsSHM1 and acts as a “guardian of autophagy” by suppressing SHM1 degradation through autophagy, thereby preserving OsSHM1 protein stability and maintaining serine metabolic homeostasis. Haplotype analysis identified a superior haplotype of *NLSS3*, Hap1, which is predominant in *japonica* accessions and correlates with higher expression and enhanced salt tolerance. Its introgression into the elite indica cultivar 9311 markedly boosts salinity resilience without agronomic penalty. Our findings reveal a novel EXO70‐SHM1 regulatory axis that integrates vesicular trafficking, autophagy, and primary metabolism, thereby offering both a genetic tool and a conceptual framework for synergistically improving plant architecture and stress tolerance in rice.

## 2 Results

### 2.1 Phenotypic Characterization of the *nlss3* Mutant

**FIGURE 1:**
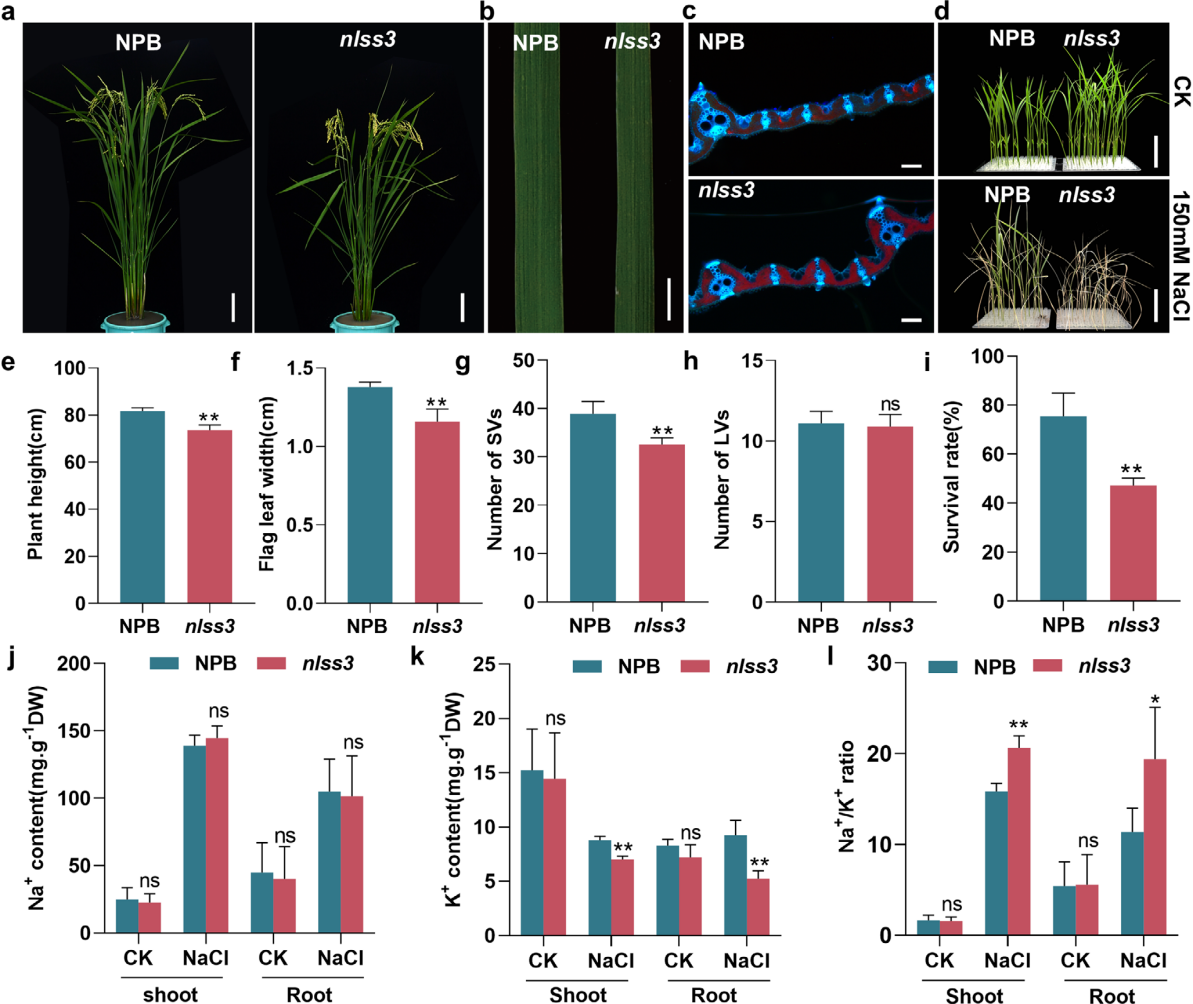
Phenotypic comparison of NPB and *nlss3*. (a, b) Representative images of NPB and *nlss* *3* plants (a) and flag leaf (b) at heading stage. Scale bars, 10 cm (a),1 cm (b). (c) Cross sections of flag leaf in NPB and *nlss3* at heading stage. Scale bars, 200 µm. (d) Performance of NPB and *nlss3* before and after salt stress at the seedling stage. Scale bars, 5 cm. (e, f) Comparative measurements of plant height (e), leaf width (f) in NPB and *nlss3*. Data represent means ± SD (*n* = 10). (g,h) Comparative measurements of the number of small vascular bundles (SVs) (g) and the number of large vascular bundles (LVs) (h) of NPB and *nlss3*. Data represent means ± SD (*n* = 10). (i) Survival rates of NPB and *nlss3* under 150 mM NaCl stress. Data represent means ± SD (*n* = 3). (j–l) Comparative measurements of Na^+^ content (j), K^+^ content (k), and Na^+^/K^+^ ratio (l) before and after salt stress in NPB and *nlss3*. Data represent means ± SD (*n* = 6). The significance of all the above data was determined by Student's *t*‐test. ^**^ for *p* < 0.01; ^*^ for *p* < 0.05; ns, not significant.

*nlss3* was obtained through EMS mutagenesis of the rice cultivar Nipponbare (NPB). In comparison to NPB, the *nlss3* mutant manifested reduced plant height, narrower leaves, and exhibited salt sensitivity during the seedling stage (Figure 1a–d). Specifically, the plant height of *nlss3* was 74.4 cm, and the width of the flag leaf was 1.16 cm, both of which were significantly lower than those of NPB (Figure 1e,f). Cross‐section analysis of the flag leaf revealed there was no difference in the number of large vascular bundles between *nlss3* and NPB; however, the number of small vascular bundles in *nlss3* was reduced by 16% (Figure 1g,h). These results indicated that the decreased number of small vascular bundles was contributing to the narrower leaf phenotype of *nlss3*. Consistent with the leaf phenotype, stems of the *nlss3* mutant were also thinner due to a reduced number of small vascular bundles (Figure S1a–d), suggesting a fundamental role for *NLSS3* in vascular development. We subjected *nlss3* to 150 mm NaCl stress at the seedling stage. Compared with NPB, *nlss3* showed a lower survival rate under salt stress, with more severe damage to growth (Figure 1i and Figure S2a–c). Maintaining Na^+^/K^+^ homeostasis is a key determinant of salt tolerance [40, 41]. Accordingly, we measured ion contents and found the *nlss3* mutant exhibited lower K^+^ accumulation and a higher Na^+^/K^+^ ratio than NPB under salt stress (Figure 1j–l). Furthermore, *nlss3* displayed an impaired ROS (Reactive oxygen species) scavenging capability under salt stress (Figure S2d–f). To further pinpoint the reason for the altered K^+^ levels under salt stress in *nlss3*, we measured the expression level of major K^+^ uptake genes, finding that it was significantly suppressed in the *nlss3* mutant under salt stress (Figure S3a–d). These results indicated that *nlss3* impairs both ion homeostasis and ROS scavenging under salt stress.

Leaf morphology is closely linked to photosynthetic efficiency. Photosynthetic parameter analysis at the heading stage revealed that *nlss3* exhibited a significantly lower photosynthetic rate and a higher intercellular CO_2_ concentration compared to NPB, while no significant differences were observed in stomatal conductance or transpiration rate (Figure S4a–d). Measurements of yield‐related traits revealed significant decreases in the number of secondary branches, grains per panicle, grain width, thousand‐grain weight, and yield per plant in *nlss3* (Figure S5a,c–h). Grain morphological analysis demonstrated a significant reduction in grain width in *nlss3*, with no observable alteration in grain length (Figure S5b,i,j). Scanning electron microscopy further revealed a pronounced decrease in the cell width of both inner and outer epidermis of the glume in *nlss3* compared to the wild type (Figure S5k,l). These results demonstrated that *nlss3* has pleiotropic effects on rice development.

### 2.2 Map‐Based Cloning and Functional Characterization of *NLSS3*

**FIGURE 2:**
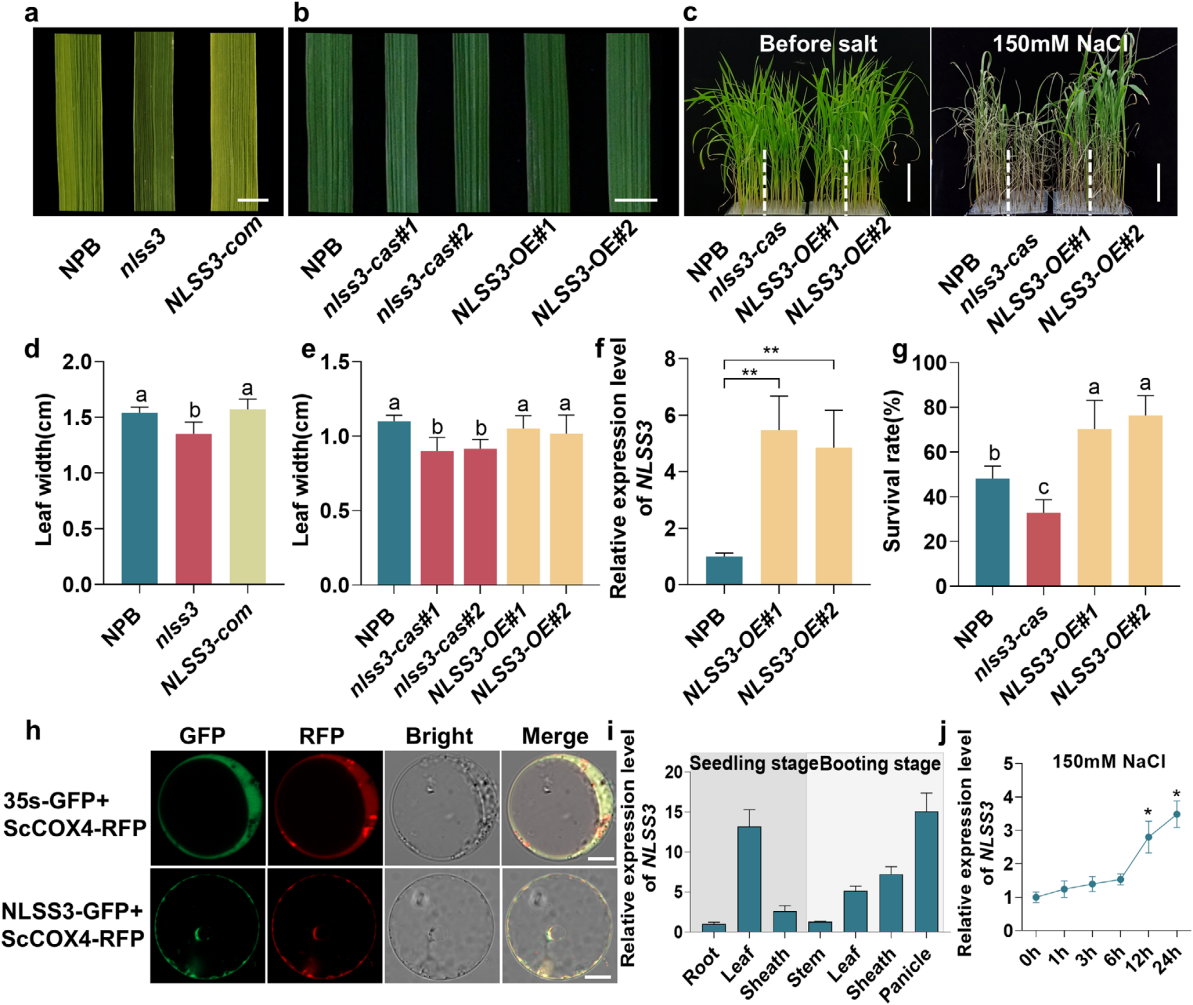
Cloning and functional characterization of *NLSS3*. (a) Representative images of the widest part of the flag leaf in NPB, *nlss3*, and *NLSS3*‐com plants at heading stage. Scale bars, 1 cm. (b) Representative images of the widest part of flag leaf in NPB, *NLSS3* knockout lines (*nlss3‐cas#1*, *nlss3‐cas#2*) and *NLSS3* overexpression lines (*NLSS3‐OE#1*, *NLSS3‐OE#2*) at tillering stage. Scale bars, 1 cm. (c) Representative images of NPB and *NLSS3* knockout and overexpression lines before and after salt stress at the seedling stage. Scale bars, 5 cm. (d) Leaf width of NPB, *nlss3*, and *NLSS3*‐com plants. Data represent means ± SD (*n* = 10). Significance was determined by one‐way ANOVA with Tukey's test. Significant differences between groups are marked with different letters. (e) Leaf width of NPB, *NLSS3* knockout lines, and overexpression lines. Data represent means ± SD (*n* = 10). Significance was determined by one‐way ANOVA with Tukey's test. Significant differences between groups are marked with different letters. (f) Relative expression levels of *NLSS3* in *NLSS3* overexpression lines at the tillering stage. Data represent means ± SD (*n* = 3). Significance was determined by Student's *t*‐test. ^**^ for *p* < 0.01. (g) Survival rates of NPB, *NLSS3* knockout, and overexpression lines under 150 mm NaCl stress. Data represent means ± SD (*n* = 3). Significance was determined by one‐way ANOVA with Tukey's test. Significant differences between groups are indicated by different letters. (h) Subcellular localization of NLSS3‐GFP fusion protein in rice protoplasts. ScCOX4‐RFP was used as the mitochondria marker. Scale bars, 10 µm. (i) Relative expression levels of *NLSS3* in different tissues of NPB plants. Data represent means ± SD (*n* = 3). (j) Relative expression levels of *NLSS3* under salt stress. Data represent means ± SD (*n* = 3). Significance was determined by Student's *t*‐test. ^*^ for *p* < 0.05.

Genetic analysis revealed that the narrow leaf phenotype of *nlss3* is controlled by a single recessive nuclear gene (Table S1). Employing map‐based cloning techniques, we identified a single nucleotide mutation (C to A) at the 395th base within *LOC_Os03g33520*. This mutation led to an amino acid substitution, specifically from alanine to proline (Figure S6). Consequently, we hypothesized that *LOC_Os03g33520* might be the gene responsible for the phenotypic traits observed in *nlss3*. To validate this hypothesis, a functional complementation assay was performed by introducing the full‐length genomic sequence of *LOC_Os03g33520* into the *nlss3* mutant. The transgenic *NLSS‐com* plant exhibited a leaf width comparable to NPB, demonstrating that *LOC_Os03g33520* is the causal gene for narrow leaf in *nlss3* (Figure 2a,d).

To further validate the function of *NLSS3*, we generated knockout and overexpression transgenic lines of *NLSS3* in the NPB background (Figure 2f and Figure S7b). Phenotypic analysis at the tillering stage revealed that the leaf width of the knockout lines was significantly narrower than that of NPB, while *NLSS3‐OE* plants showed no difference in leaf width (Figure 2b,e). Cross‐section analysis indicated that this reduction was primarily attributed to a decrease in the number of small vascular bundles, rather than large vascular bundles (Figure 2e and Figure S7a,c,d). Under salt stress treatment, the *NLSS3* knockout lines exhibited a significantly reduced survival rate, whereas the overexpression lines showed enhanced tolerance compared NPB (Figure 2c,g). Collectively, these results demonstrated that *NLSS3* positively regulates salt tolerance and is essential for normal leaf development in rice.

Subcellular localization analysis indicated that NLSS3 localizes to mitochondria, as evidenced by its co‐localization with the mitochondrial marker protein ScCOX4 (Figure 2h) [42]. Further qRT‐PCR analysis showed that it was mainly expressed in seedling leaves, as well as in the sheath, leaves, and panicles of mature plants (Figure 2i), and was significantly induced under salt stress (Figure 2j). To further verify the qRT‐PCR results, we employed the β‐glucuronidase (GUS) reporter system to visualize *NLSS3* expression, which yielded patterns consistent with the qRT‐PCR data (Figure S8a). For higher spatial resolution, we performed GUS staining and RNA in situ hybridization on leaf primordia. These analyses revealed that *NLSS3* signals are concentrated in all vascular bundles (Figure S8b,c). Consistent with this, qRT‐PCR confirmed high *NLSS3* expression in both small and large vascular bundles (Figure S8d). Taken together, these data collectively demonstrated vascular expression of *NLSS3*, which is consistent with its role in regulating leaf width.

### 2.3 NLSS3 Interacts With SHM1 and Attenuates Its Autophagic Degradation

**FIGURE 3:**
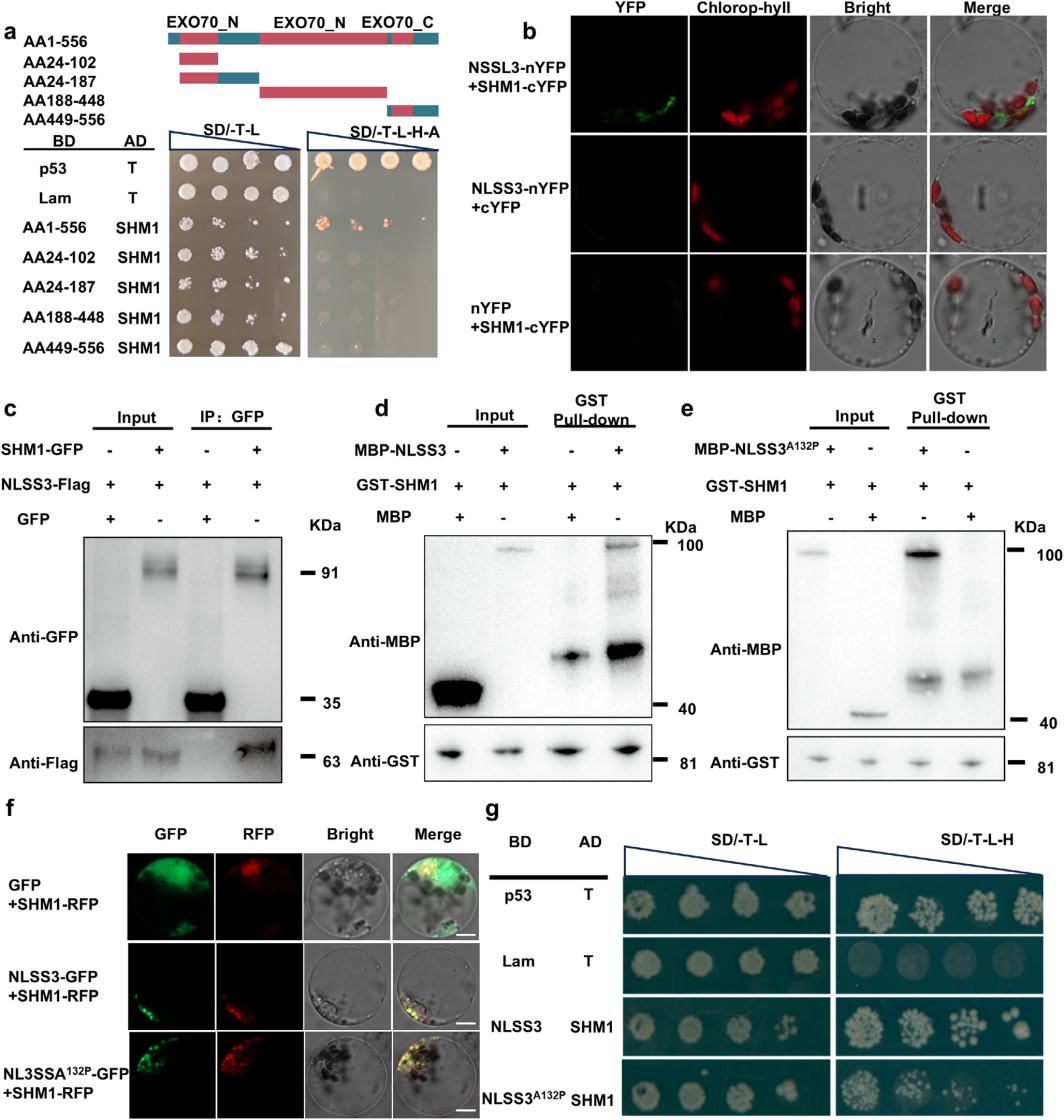
NLSS3 directly interacts with SHM1, and the A132P mutation weakens this interaction. (a) Yeast two‐hybrid assay shows full length of NLSS3 can interact with SHM1. (b) BiFC assay in rice protoplasts showing NLSS3‐SHM1 interaction. (c) Co‐IP validating NLSS3‐SHM1 interaction in rice protoplasts. NLSS3‐Flag and SHM1‐GFP were co‐transfected into rice protoplasts. The approximate positions of the protein molecular weight markers are labeled on the right side. (d) Pull‐down assays showed the physical interaction of NLSS3 and SHM1. GST‐SHM1, Maltose‐binding protein (MBP), and MBP‐NLSS3 were purified with pull‐down assays and detected with anti‐MBP antibodies (MBP and MBP‐NLSS3) and anti‐GST antibodies (GST‐SHM1). The approximate positions of the protein molecular weight markers are labeled on the right side. (e) Pull‐down assay testing NLSS3^A132P^‐SHM1 interaction. The approximate positions of the protein molecular weight markers are labeled on the right side. (f) Co‐localization of NLSS3/NLSS3^A132P^ with SHM1 in rice protoplasts. Scale bars, 10 µm. (g) Yeast two‐hybrid quantification showing interaction strength between NLSS3‐SHM1 and NLSS3^A132P^‐SHM1.

To elucidate the molecular mechanism by which *NLSS3* regulates rice leaf development and salt stress tolerance, we performed a yeast two‐hybrid library screening approach to identify proteins interacting with NLSS3. SHM1, encoding a key enzyme involved in the plant photorespiration pathway, directly interacted with NLSS3. Truncated NLSS3 variants failed to interact with SHM1 in yeast two‐hybrid assays (Figure 3a). Further validation through bimolecular fluorescence complementation (BiFC), co‐immunoprecipitation (Co‐IP), and pull‐down experiments confirmed the direct interaction between NLSS3 and SHM1 both in vivo and in vitro (Figure 3b–d). These results indicated that NLSS3 did interact with SHM1, and that every functional segment of NLSS3 is essential for its interaction with SHM1. This led us to hypothesize whether the NLSS3^A132P^ point mutation in the *nlss3* mutant affects its direct interaction with SHM1. To test this hypothesis, we first investigated the co‐localization of both NLSS3 and NLSS3^A132P^ with SHM1. The results showed that the NLSS3^A132P^ mutation did not alter their co‐localization pattern (Figure 3f). Subsequently, pull‐down assays confirmed the direct interaction between NLSS3^A132P^ and SHM1 (Figure 3e). However, yeast two‐hybrid results demonstrated that the NLSS3^A132P^ mutation significantly weakened its interaction with SHM1 (Figure 3g). Similarly, Split‐luciferase assays in tobacco further validated that the A132P mutation in NLSS3 weakens its interaction with SHM1 (Figure S9a,b).

**FIGURE 4:**
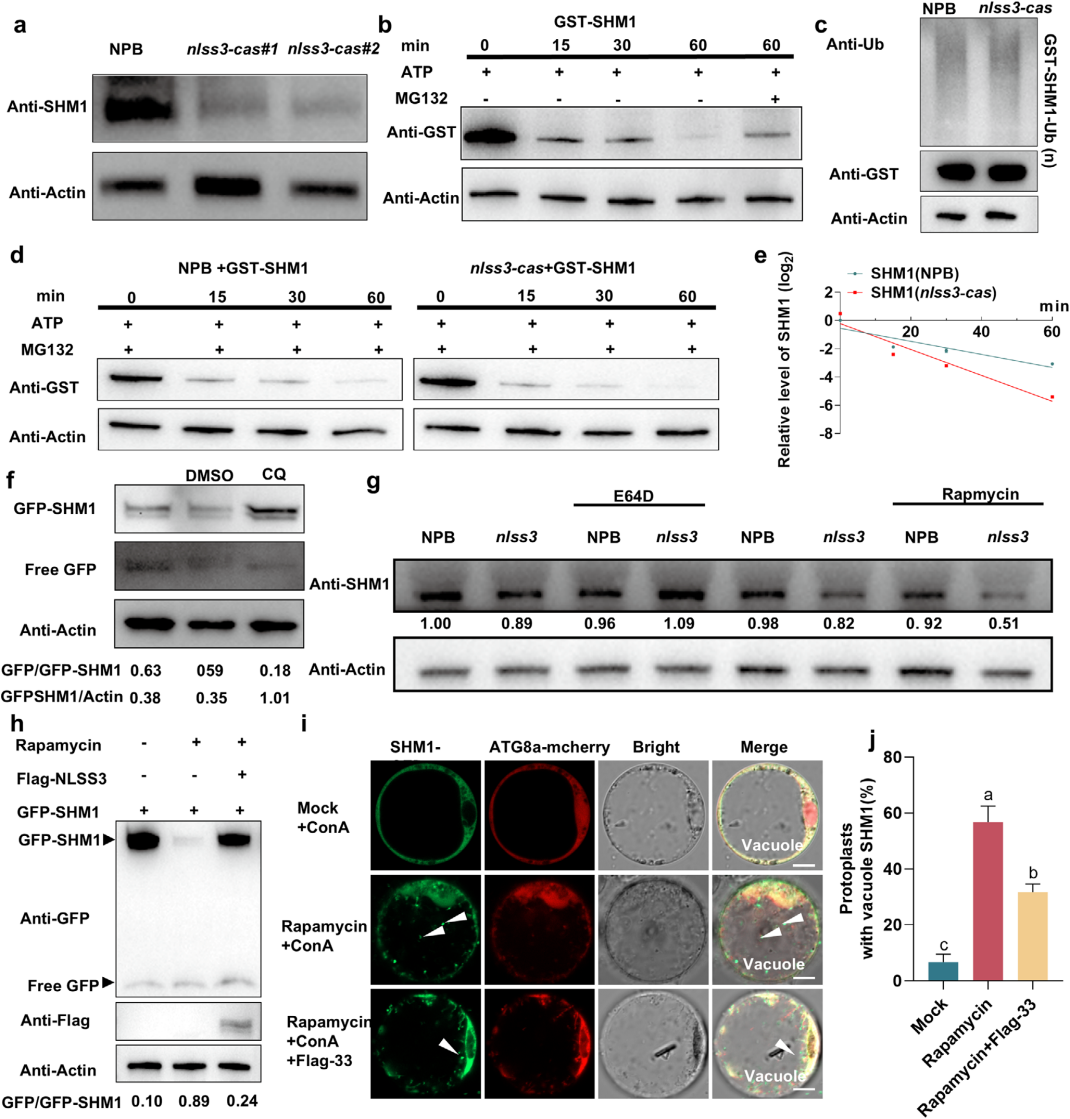
NLSS3 suppresses autophagic degradation of SHM1. (a) Comparison of SHM1 protein abundance in NPB and *nlss3‐cas* plants at the seedling stage. (b) SHM1 degradation assay in NPB seedling extracts with or without MG132 treatment. (c) Detection of SHM1 ubiquitination levels in NPB and *nlss3‐cas* at the seedling stage using ubiquitin antibody. (d) In vitro degradation assay of GST‐SHM1 in NPB and *nlss3‐cas* plants at the seedling stage. (e) Protein abundance fitting curve of the in vitro degradation assay. (f) Effect of autophagy inhibitor CQ treatment on GFP‐SHM1 level in protoplasts. (g) Effect of autophagy inhibitor E64D and inducer rapamycin on SHM1 protein level in NPB and *nlss3* at the seedling stage. (h) Effect of NLSS3 on SHM1 autophagy in protoplasts. (i) Subcellular localization of SHM1 under normal and autophagy‐induced conditions with or without NLSS3. Arrows indicate GFP‐SHM1 puncta inside the vacuole. Scale bars, 10 µm. (j) Rate of SHM1 puncta inside the vacuole in protoplasts with or without NLSS3. Data represent means ± SD (*n* = 3). Significance was determined by one‐way ANOVA with Tukey's test. Significant differences between groups are indicated by different letters.

Based on the established role of protein‐protein interactions in stabilizing complex components, we hypothesized that NLSS3 may regulate SHM1 stability. Consistent with this hypothesis, SHM1 protein abundance was markedly reduced in *nlss3‐cas* plants (Figure 4a). Given previous reports of ubiquitin‐mediated SHM1 degradation [37], we initially investigated whether NLSS3 affects SHM1 stability through the ubiquitin proteasome pathway. While MG132 treatment effectively stabilized SHM1 (Figure 4b), comparable levels of SHM1 ubiquitination were observed in NPB and *nlss3‐cas* plant (Figure 4c). Furthermore, SHM1 degradation was markedly accelerated in *nlss3‐cas* extracts even in the presence of the proteasome inhibitor MG132 (Figure 4d,e). These results indicated that NLSS3 stabilizes SHM1 independent of the ubiquitin pathway, implicating a ubiquitin‐independent proteolytic pathway.

Selective autophagy represents a major route for targeted protein turnover distinct from proteasomal degradation; we suspected NLSS3 could regulate SHM1 stability through this mechanism. Free GFP assay, which utilizes the differential stability of GFP‐fusion proteins upon vacuolar delivery (where the fused protein is rapidly degraded while the free GFP accumulates), provides a reliable measure of autophagic activity. The presence and relative abundance of free GFP thus serve as indicators of autophagy‐mediated degradation and flux intensity [43]. Treatment with the autophagy inhibitor chloroquine (CQ) significantly increased SHM1 protein abundance and reduced the free GFP/GFP‐SHM1 ratio in protoplasts (Figure 4f). Consistent with this, pharmacological assays in plants showed that the SHM1 deficiency in *nlss3* plant was alleviated by the autophagy inhibitor Aloxistatin (E64D) but aggravated by the autophagy inducer Rapamycin (Figure 4g). Furthermore, a cell‐free degradation assay revealed that the rate of SHM1 degradation was comparable between NPB and the *nlss3‐cas* under CQ treatment (Figure S10a,b). These results indicated that SHM1 can be degraded via autophagy, and that NLSS3 acts as a negative regulator of SHM1 autophagic degradation.

To further investigate the role of NLSS3 in the autophagic degradation of SHM1, we co‐expressed Flag‐NLSS3 with GFP‐SHM1 in rice protoplasts. Notably, under Rapamycin‐induced autophagy conditions, co‐expression of NLSS3 led to a significant decrease in the free GFP/GFP‐SHM1 ratio and enhanced SHM1 protein stability compared with the expression of GFP‐SHM1 alone. (Figure 4h). Treatment with Rapamycin and Concanamycin A (ConA) strongly induced the formation of GFP‐SHM1 puncta in the vacuole, whereas co‐expression of Flag‐NLSS3 markedly attenuated this induction (Figure 4i,j). Salt stress is a common inducer of autophagy in plants [44]. We detected SHM1 protein levels in *NLSS3‐OE* lines before and after salt treatment. The results showed that *NLSS3* overexpression enhanced SHM1 accumulation under salt stress, indicating that NLSS3 attenuates SHM1 degradation via autophagy (Figure S10c). These findings collectively demonstrated that NLSS3 interacts with SHM1 and specifically attenuates its autophagic degradation, particularly under autophagy‐inducing conditions such as salt stress.

### 2.4 Loss of SHM1 Function in *nlss3* Compromised Plant Growth and Salt Stress Tolerance

**FIGURE 5:**
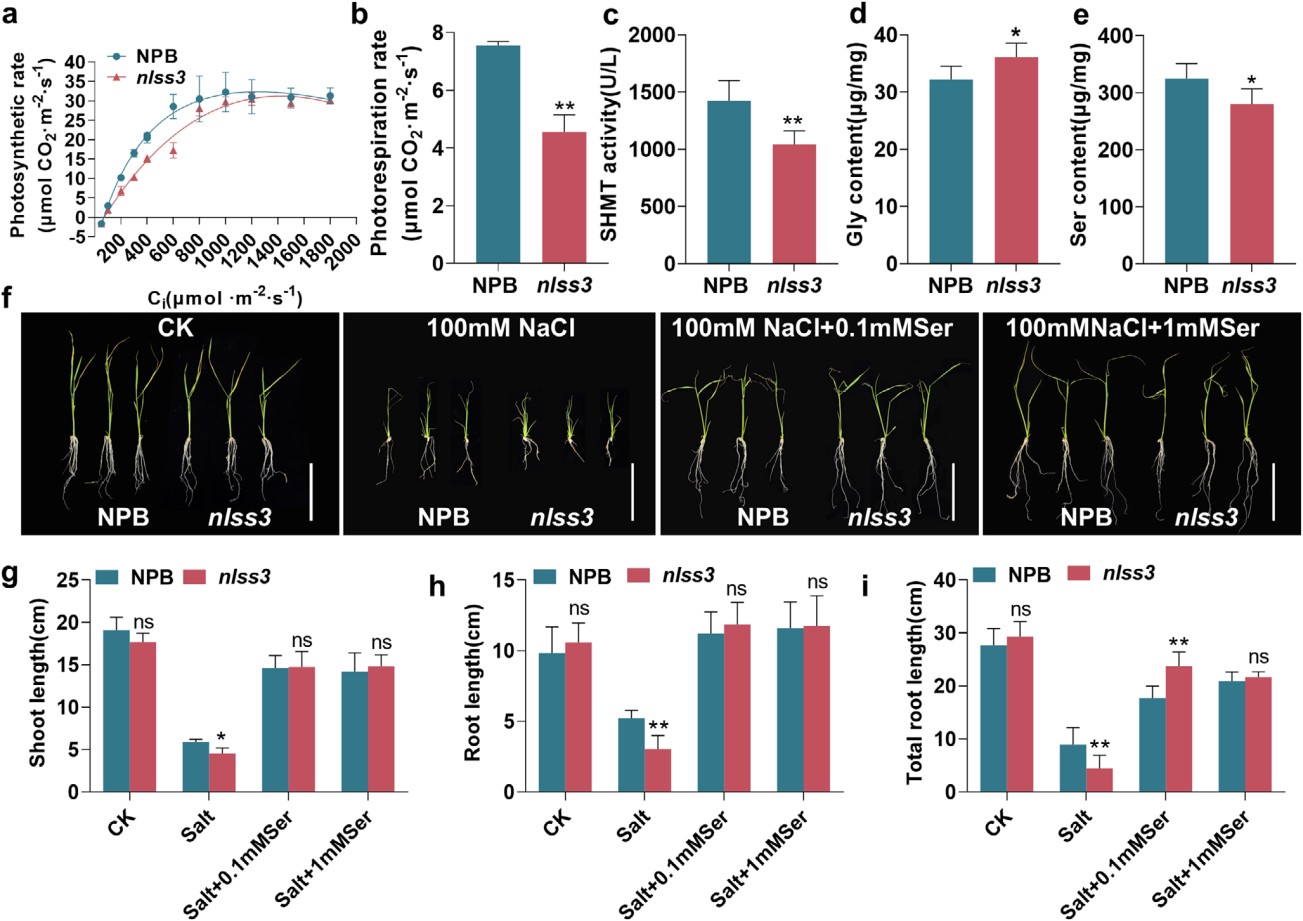
Downregulation of SHM1 in *nlss3* impairs salt resistance. (a, b) Photorespiration rates of NPB and *nlss3* determined by fitting CO_2_ response curves at the tillering stage, CO_2_ response curves (a), and photorespiration rates (b). Data represent means ± SD (*n* = 3). (c) SHMT enzyme content of NPB and *nlss3* at the seedling stage. Data represent means ± SD (*n* = 5). (d) Glycine content of NPB and *nlss3* at the seedling stage. Data represent means ± SD (*n* = 5). (e) Serine content in NPB and *nlss3* at the seedling stage. Data represent means ± SD (*n* = 5). (f) Phenotypes of NPB and *nlss3* seedlings under salt stress with or without serine supplementation. Scale bars, 10 cm. (g–i) Growth parameters of NPB and *nlss3* seedlings under different serine concentrations at the seedling stage. shoot length (g), root length (h), total root length (i). Data represent means ± SD (*n* > 5). The significance of all the above data was determined by Student's *t*‐test, ^**^ for *p *< 0.01; ^*^ for *p* < 0.05; ns, not significant.

*SHM1* encodes serine hydroxymethyltransferase (SHMT), a key enzyme that catalyzes the conversion of glycine to serine during the terminal step of the photorespiratory pathway. Given our finding that NLSS3 is required for the stabilization of SHM1, we hypothesized that the developmental and stress‐related phenotypes observed in *nlss3* may stem from compromised SHMT activity. To test this hypothesis, we first assessed photorespiration rates in NPB and *nlss3* through CO_2_ response curves [45]. The results revealed a significant reduction in photorespiration rate in *nlss3* compared to NPB (Figure 5a,b). Furthermore, the *nlss3* mutant exhibited reduced SHMT activity, along with glycine accumulation and decreased serine levels (Figure 5c–e). Broader metabolomics confirmed a decrease in photorespiratory intermediates (2‐phosphoglycolate,2‐oxoglutarate, serine) and an increase in glycine (Figure S11a), whereas the activities of hydroxypyruvate reductase (HPR) and the glycine decarboxylase complex (GDC) were unaffected (Figure S11b,c). Thus, *nlss3* impairs photorespiratory flux primarily by disrupting SHMT function.

To determine whether the phenotypic defects in *nlss3* are associated with reduced serine levels, we performed salt stress treatment with or without serine supplementation. Under salt stress, both NPB and *nlss3* showed inhibited growth in shoots and roots, with more severe impairment in *nlss3* seedlings (Figure 5f). However, when treated with exogenous serine, the salt‐sensitive phenotype of *nlss3* was markedly rescued, as evidenced by comparable shoot length, root length, and total root length to NPB (Figure 5g–i). These findings confirmed that the salt sensitivity of *nlss3* is primarily caused by serine deficiency resulting from SHM1 dysfunction.

### 2.5 *SHM1* Acted Downstream of *NLSS3* in Regulating Leaf Width and Salt Tolerance

**FIGURE 6:**
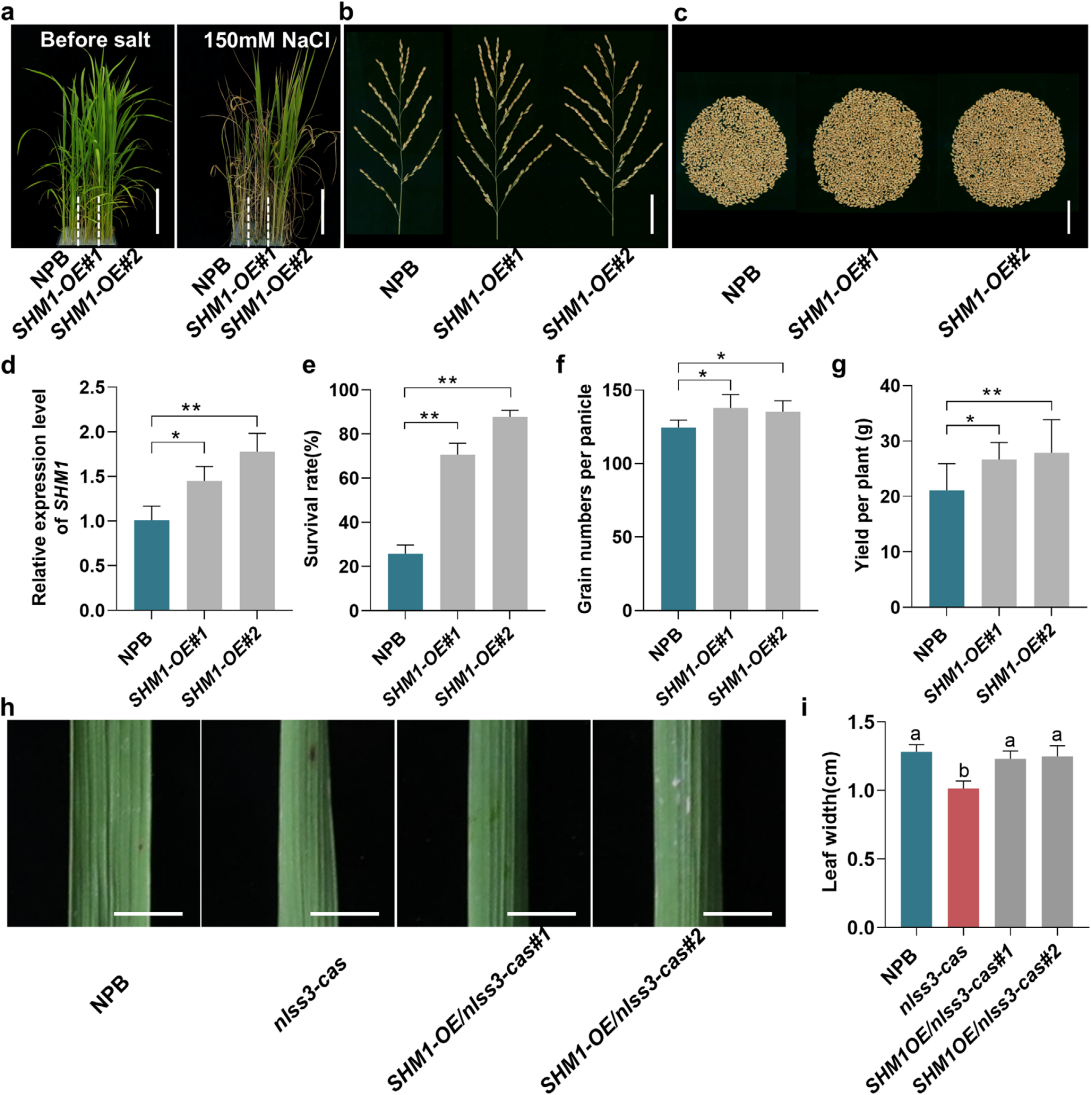
*SHM1* acted downstream of *NLSS3* to regulate growth and salt stress tolerance in rice. (a) Phenotypes of NPB and *SHM1* overexpression lines (*SHM1‐OE#1*, *SHM1‐OE#2*) before and after salt stress treatment at the seedling stage. Scale bars, 10 cm. (b) Panicle image of NPB and *SHM1* overexpression lines. Scale bars, 5 cm. (c) Grains per plant image of NPB and *SHM1* overexpression lines. Scale bars, 5 cm. (d) Relative expression levels of *SHM1* in NPB and *SHM1* overexpression lines at the tillering stage. Data represent means ± SD (*n* = 3). Significance was determined by Student's *t*‐test, ^*^ for *p* < 0.05, ^**^ for *p* < 0.01. (d) Survival rates of NPB and *SHM1* overexpression lines under salt stress. Data represent means ± SD (*n* = 3). Significance was determined by Student's *t*‐test, ^**^ for *p* < 0.01. f) Grain numbers per panicle of NPB and *SHM1* overexpression lines. Data represent means ± SD (*n* = 10). Significance was determined by Student's *t*‐test, ^*^ for *p* < 0.05. (g) Yield per plant of NPB and *SHM1* overexpression lines. Data represent means ± SD (*n* = 10). Significance was determined by Student's *t*‐test, ^**^ for *P* < 0.01. (h) Leaf images of NPB, *NLSS3* knockout mutant, and *SHM1OE/nlss3‐cas* plants at the heading stage. Scale bars, 1 cm. (i) Leaf width of NPB, *NLSS3* knockout mutant, and *SHM1OE nlss3‐cas* plants. Data represent means ± SD (*n* = 5). Significance was determined by one‐way ANOVA with Tukey's test. Significant differences between groups are indicated by different letters.

To elucidate the role of *SHM1* in plant growth and stress tolerance, we generated *SHM1* knockout mutants in the NPB background using gene editing. Consistent with previous reports, the *shm1* exhibited chlorosis and lethality after the three‐leaf stage [38]. We also generated *SHM1* overexpression lines in the NPB background (Figure 6d). When subjected to salt stress, these overexpression lines displayed markedly enhanced salinity tolerance compared with NPB (Figure 6a,e). Considering that the enhancement of resistance is frequently accompanied by yield penalties, the agronomic traits of *SHM1* overexpression plants were also evaluated (Figure 6b,c). Notably, rather than suppressing plant growth, the overexpression of *SHM1* led to a significant increase in grain numbers per panicle and yield per plant (Figure 6f,g). Detailed phenotypic analysis revealed that while the *SHM1* overexpression plants exhibited no alterations in panicle length, seed setting rate, or secondary branch numbers, they exhibited a significant increase in effective panicle numbers, primary branch number, which resulted in a higher grain number per panicle and enhanced yield per plant (Figure S12a–g).

To determine the genetic relationship between *NLSS3* and *SHM1*, we generated *SHM1* overexpression constructs in the *nlss3‐cas* mutant background. Notably, ectopic expression of *SHM1* fully rescued SHM1 deficiency and the narrow leaf phenotype of *nlss3‐cas* plant (Figure 6h,i and Figure S13a). Similarly, SHM1 protein levels were restored in *NLSS3‐com* plants (Figure S13b). Taken together, these results indicated that SHM1 protein abundance is strongly correlated with the phenotype of the *nlss3* mutant and *SHM1* acts downstream of *NLSS3* in a regulatory pathway that integrates developmental control and environmental stress adaptation.

### 2.6 The Favorable *NLSS3* Haplotype From *Japonica* Could Enhance Rice Salt Tolerance

**FIGURE 7:**
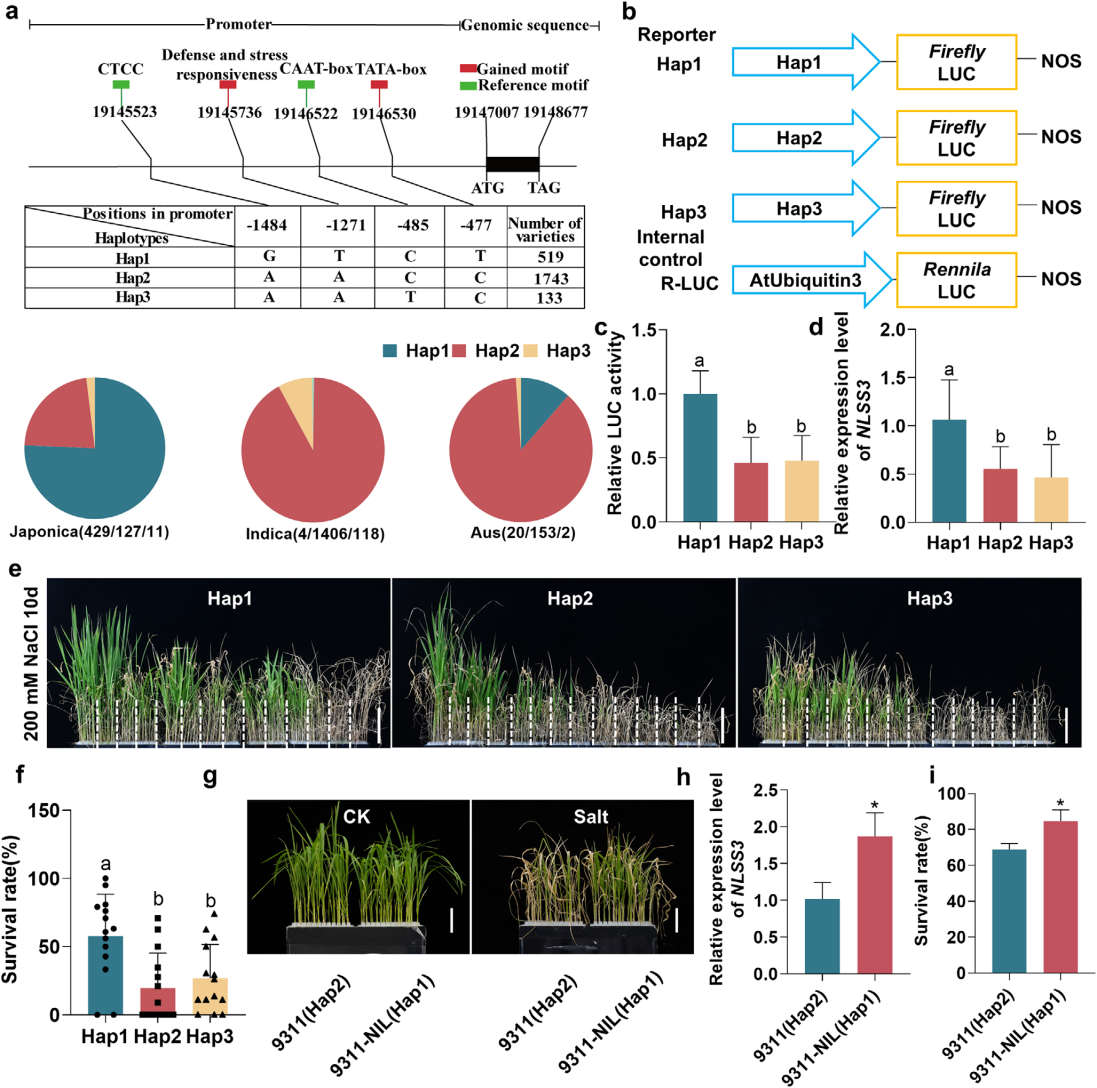
The favorable haplotype of *NLSS3* enhanced salt tolerance in rice. (a) Three major *NLSS3* haplotypes based on promoter SNPs, showing distinct distributions among subpopulations. (b,c) Differential promoter activities of *NLSS3* haplotypes measured by LUC reporter assay. Data represent means ± SD (*n* = 5). Significance was determined by one‐way ANOVA with Tukey's test. Significant differences between groups are indicated by different letters. (d) Relative expression levels of *NLSS3* in accessions representing different haplotypes at the seedling stage. Data represent means ± SD (*n* = 10). Significance was determined by one‐way ANOVA with Tukey's test. Significant differences between groups are indicated by different letters. (e) Phenotypes of representative accessions from each haplotype under 200 mm NaCl treatment at the seedling stage. Scale bars, 10 cm. (f) Survival rates of accessions from each haplotype under 200 mm NaCl stress. Data represent means ± SD (*n* = 14). Significance was determined by one‐way ANOVA with Tukey's test. Significant differences between groups are indicated by different letters. (g) Phenotypes of 9311 (Hap2) and 9311‐NIL (Hap1) before and after salt stress at the seedling stage. Scale bars, 5 cm. (h) Relative expression of *NLSS3* in 9311 (Hap2) and 9311‐NIL (Hap1) at the seedling stage. Data represent means ± SD (*n* = 3). Significance was determined by Student's *t*‐test. ^*^ for *P* < 0.05. (i) Survival rates of 9311 (Hap2) and 9311‐NIL (Hap1) under salt stress. Data represent means ± SD (*n* = 3). Significance was determined by Student's *t*‐test. ^*^ for *P* < 0.05.

Haplotype analysis of *NLSS3* using the 3K rice genome database (https://snp‐seek.irri.org/) identified four key SNPs (single‐nucleotide polymorphisms) in the promoter region, which were classified into three major haplotypes: Hap1 (GTCT), Hap2 (AACC), Hap3 (AATC). Analysis of the distribution of these haplotypes in major subpopulations revealed that Hap1 is predominantly present in the *japonica* accessions, while Hap2 is mainly found in the *indica* and *aus* accessions (Figure 7a). Given that promoter variations are often associated with gene expression levels, we assessed the promoter activity of different haplotypes using LUC (Luciferase) reporter system. The results showed Hap1 exhibiting the highest activity (Figure 7b,c), which was consistent with the expression pattern of *NLSS3* in the corresponding varieties (Figure 7d). These findings suggest that phenotypic variation among haplotypes may primarily stem from differences in *NLSS3* expression.

To investigate the functional implications of these haplotypes in salt stress response, we selected 14 representative accessions for each haplotype and subjected them to salt stress at the seedling stage. Phenotypic evaluation revealed that Hap1‐carrying accessions displayed superior salt tolerance, as evidenced by markedly higher survival rates under saline conditions relative to those harboring Hap2 or Hap3 (Figure 7e,f). To further validate the causal role of the *NLSS3* haplotype in salt tolerance, we developed a near‐isogenic line (NIL) by introgressing the NPB (Hap1) allele into the 9311 (Hap2) genetic background, hereafter designated 9311‐NIL (Hap1). 9311‐NIL (Hap1) exhibited upregulated *NLSS3* expression and significantly enhanced salt tolerance (Figure 7g–i), along with elevated SHM1 activity and protein levels under salt stress (Figure S14a,b). Notably, this improvement in stress resilience was achieved without adverse effects on key agronomic traits (Figure S15a–h), underscoring the breeding value of this allele. Collectively, these results demonstrate that the favorable haplotype of *NLSS3* can improve rice salt tolerance without compromising yield.

## 3 Discussion

The EXO70 protein, an integral constituent of the exocyst complex, exerts substantial influence on plant growth, development, and responses to biotic stresses [28, 29, 30, 46, 47]. However, its function and regulatory mechanisms in abiotic stress responses, such as salt stress, remain unclear. This study identified a novel gene encoding the EXO70 protein, *NLSS3*. The *NLSS3* mutation resulted in narrowed leaves, which aligns with the reported role of EXO70 family members in regulating vascular development [48, 49]. Additionally, a few EXO70 members have been implicated in autophagy regulation [26, 50]; however, the underlying mechanisms remain incompletely understood. This study reveals a novel mechanism by which EXO70 proteins participate in autophagy regulation. Unlike the established role of EXO70D as an autophagy receptor [27], NLSS3 functions as a “guardian of autophagy” by binding to and stabilizing SHM1, thereby inhibiting its autophagic degradation. These findings extend beyond the current understanding of EXO70 protein functions and provide new insights into the functional diversity of the EXO70 family as well as the complexity of the autophagy regulatory network. However, the molecular mechanism by which NLSS3 binds to SHM1 and maintains its autophagic balance requires further investigation. Our Transmission Electron Microscope (TEM) observations revealed that the number of autophagosomes remained unchanged in *nlss3* root cells compared to NPB, arguing against a role for NLSS3 in globally regulating autophagosome formation (Figure S16a,b). Therefore, we propose that NLSS3 stabilizes SHM1 through a competitive binding mechanism, shielding SHM1 from recognition by a specific autophagy receptor and thereby diverting it from selective autophagic degradation. Intriguingly, analogous receptor‐competition mechanisms that fine‐tune protein homeostasis via autophagy flux have been reported in diverse biological contexts, such as antiviral immunity in mice and leaf senescence in plants, suggesting a conserved regulatory strategy [51, 52]. In line with this model, we found that overexpressing *NLSS3* specifically enhances SHM1 abundance under salt stress (an autophagy‐inducing condition) but not under normal growth conditions, indicating that NLSS3 precisely regulates SHM1 stability in an autophagy‐dependent manner.

*SHM1*, which encodes a key enzyme in the photorespiratory pathway, is of paramount importance not only in carbon and nitrogen metabolism but also in maintaining ROS homeostasis. Thus, the precise regulation of SHM1 is indispensable for striking a balance between plant growth and stress adaptation. This study validates that the overexpression of *SHM1* significantly enhances salt tolerance and boosts yield in rice, underscoring its dual function in positively regulating both abiotic stress resistance and growth. Previous research has indicated that SHM1 is subject to complex post‐transcriptional regulation. In Arabidopsis, SHM1 stability is positively regulated by FER‐mediated phosphorylation and UBP16‐mediated deubiquitination, which in turn enhances salt tolerance [53]. Conversely, under pathogen stress, the rice protein MEL modulates ROS levels by promoting the ubiquitination‐mediated degradation of SHM1, subsequently activating disease resistance responses [37]. This study enriches the regulatory network by demonstrating that SHM1 can be degraded via the autophagy pathway, while NLSS3 acts as an “autophagy guardian” to specifically inhibit this process, thereby preserving growth and salt stress tolerance. These findings suggest that SHM1 stability serves as a crucial node integrating diverse stress signals and is precisely regulated by multiple pathways, including ubiquitination and autophagy. Future research should clarify the mechanism by which SHM1 is recognized by autophagy and the molecular details of how NLSS3 precisely intervenes in this process, thus comprehensively unraveling the complex network through which plants fine‐tune SHM1 homeostasis under different stress conditions. The autophagic and ubiquitination‐mediated degradation of SHM1 may represent a dual safeguard for its precise regulation in varying environments and under different stresses. Under normal growth conditions and abiotic stresses such as salt stress, NLSS3 inhibits SHM1 autophagy, and ubiquitination may also be suppressed to maintain normal development and abiotic stress resistance. In contrast, during pathogen infection, both autophagy and ubiquitination could be activated, facilitating the rapid degradation of the SHM1 protein, maintaining ROS levels, and enhancing disease resistance.

Serine serves as a critical metabolic node in living organisms, extensively participating in folate metabolism, glutathione synthesis, and the production of various biomolecules. Its homeostasis is essential for plants [54]. In plants, serine biosynthesis occurs through three distinct pathways, with the photorespiratory pathway serving as the primary source [55]. SHMT is the core enzyme catalyzing this reaction and is functionally indispensable, as evidenced by the lethality of *shm1* in both Arabidopsis thaliana and rice due to ROS accumulation, indicating that *SHM1* performs a similar and conserved function across diverse plant species [38, 56]. This study provides further support for this perspective. The *nlss3* mutant, characterized by diminished SHM1 stability, displays compromised serine biosynthesis accompanied by growth inhibition and salt sensitivity, a phenotype that is reversible upon exogenous serine application. These findings broaden the functional role of *SHM1* beyond its essential function in sustaining basal survival to include the precise regulation of abiotic stress adaptation. Nevertheless, the downstream signaling mechanisms mediated by serine fluctuation, particularly how they modulate specific developmental programs or antioxidant pathways, require further investigation.

The identification of genetic resources that simultaneously confer stress tolerance and yield potential is of utmost significance for crop genetic improvement. To address various aspects of rice production, scientists have discovered numerous superior allelic variants governing different agronomic traits. *NAL1* and *FLW7* are key determinants of leaf width in rice. Utilizing superior alleles of these genes can lead to a significant increase in grain yield [57, 58]. This study identifies a novel gene, *NLSS3*, which concurrently regulates leaf development and salt tolerance. Its superior haplotype, Hap1, is not only significantly associated with salt tolerance at the seedling stage but also does not result in a yield penalty, indicating direct application potential. This discovery provides a new genetic target for mitigating salt stress and ensuring food security. Furthermore, the overexpression of its interacting protein, SHM1, enhances salt tolerance and even increases grain yield, suggesting that SHM1 may serve as a pivotal nodal gene in the synergistic regulation of both yield and stress resistance. However, it remains to be determined whether the salt tolerance effect of *NLSS3* persists across key developmental stages such as germination and booting, and whether natural allelic variations of SHM1 possess similar application value. Addressing these questions will facilitate the effective utilization of these superior alleles in breeding programs.

## 4 Conclusion

**FIGURE 8:**
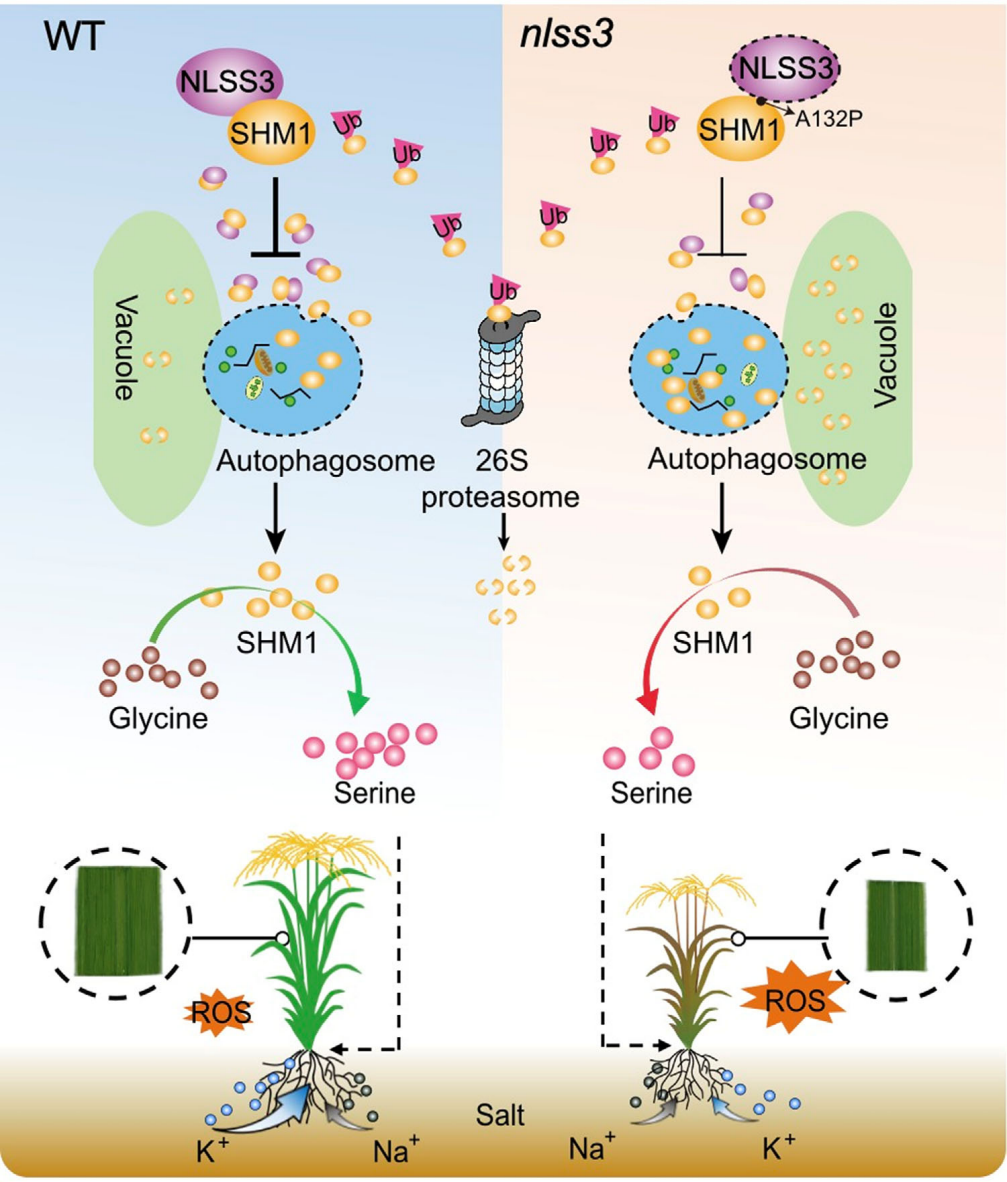
Proposed working model of NLSS3 in regulating leaf width and salt resistance. The NLSS3^A132P^ in *nlss3* has a weaken interaction affinity with SHM1 which reduces SHM1 protein level through autophagy and reduces serine content.

In summary, this study identifies *NLSS3*, a gene encoding an EXO70 family protein, as a pivotal regulator of leaf morphogenesis and salt stress tolerance in rice. We propose a mechanistic model in which NLSS3 interacts with the SHM1 to modulate its autophagic turnover, thereby safeguarding serine homeostasis. This metabolic equilibrium is crucial for normal leaf development and robust stress resilience, as it ensures adequate K^+^ homeostasis and efficient ROS scavenging. The A132P mutation in *nlss3* weakens the NLSS3‐SHM1 interaction, leading to accelerated degradation of SHM1, serine deficiency, and the consequent pleiotropic phenotypes of narrow leaves and hypersensitivity to salinity (Figure 8). Haplotype analysis reveals that Hap1, a naturally occurring allele of *NLSS3* predominantly found in japonica rice, is associated with elevated transcript abundance and enhanced salt tolerance. Notably, introgression of the Hap1 allele into the elite indica cultivar 9311 significantly improves salinity resistance without compromising agronomic performance, highlighting its breeding value.

Our findings not only uncover a previously uncharacterized role for EXO70 proteins in the integration of plant development and abiotic stress responses but also delineate a molecular framework through which NLSS3 coordinates growth and environmental adaptation by stabilizing a central metabolic enzyme. These insights advance our understanding of the intricate crosstalk between cellular metabolism and stress signaling in plants. Moreover, this work provides a valuable genetic resource and a mechanistic foundation for the molecular design of salt‐tolerant rice varieties, offering promising avenues for sustainable crop improvement in the face of escalating soil salinization.

## 5 Experimental Section

### 5.1 Plant Materials and Growth Conditions

The rice (*Oryza sativa* L) cultivar “Nipponbare” was used as the experimental material. Chemical mutagenesis was performed by treating seeds with 1% ethyl methanesulfonate (EMS) solution. Plants were cultivated in the experimental field of the China National Rice Research Institute (Fuyang, Zhejiang, 30°4′52″N, 119°55′54″E) under natural environmental conditions. Mutants exhibiting altered leaf morphology and abiotic stress responses were identified through a forward genetics screening approach.

For the assessment of salt tolerance at the seedling stage, five‐leaf‐stage seedlings were subjected to a nutrient solution containing 150 mm NaCl, with a normal nutrient solution used as the control. The treatment was maintained until significant phenotypic differences were observed.

To evaluate the effect of serine on salt stress tolerance, seedlings were grown on half‐strength Murashige and Skoog (1/2 MS) medium supplemented with 100 mM NaCl and varying concentrations of serine. Seedling growth was monitored until distinct phenotypic differences became apparent.

### 5.2 Determination of Na^+^ and K^+^ Contents

The Na^+^ and K^+^ contents were determined by flame photometry. Shoot tissues were harvested, deactivated at 105°C for 30 min, and then dried at 65°C to a constant weight. An appropriate amount of dried sample was weighed, ground into powder, and completely digested by high‐temperature digestion. The supernatant was collected after cooling and analyzed using a flame photometer (FP640, China). The actual concentrations of sodium and potassium ions were calculated based on a standard curve.

### 5.3 Histology and Microscopic Observations

Free‐hand sectioning of leaves or internodes was performed using a double blade. The resulting thin sections were immediately observed and imaged under a fluorescence microscope (Leica DM4B, Germany). For the observation of glume microstructure, scanning electron microscopy (SEM) was employed. Fresh glumes were directly placed into the sample chamber of an SEM instrument (FEI Quanta 200, USA) for direct observation of their surface ultrastructure.

For transmission electron microscopy, root tips were fixed for at least 12 h with 2.5% (w/v) glutaraldehyde. Samples were processed as previously described and observed using a transmission electron microscope (H‐7650, Japan)[31].

### 5.4 Measurement of Photosynthetic Parameters

Photosynthetic parameters of NPB and *nlss3* mutants were measured at the heading stage using a Li‐COR 6400 portable photosynthesis system.

### 5.5 Map‐Based Cloning

Map‐based cloning of the target gene was performed using an F_2_ population derived from a cross between the *nlss3* mutant and the indica cultivar 9311 and newly developed SSR and STS molecular markers (Table S2). To identify the causal mutation, genomic DNA fragments of the candidate gene were amplified from both NPB and *nlss3*, followed by sequencing and subsequent sequence alignment analysis.

### 5.6 Vector Construction and Plant Transformation

To generate the genomic DNA complementation vector for *NLSS3*, a 4673 bp genomic fragment (comprising 2000 bp upstream of the start codon, the 1673 bp coding region of *NLSS3*, and 1000 bp downstream of the stop codon) was amplified from Nipponbare (NPB) and cloned into the binary vector *pCAMBIA1300*. For the CRISPR/Cas9‐mediated knockout of *NLSS3* and *SHM1*, specific single‐guide RNA (sgRNA) target sequences were designed. The corresponding sgRNA expression cassettes (OsU3‐NLSS3 and OsU3‐SHM1) were subsequently assembled into the *pYLCRISPR/Cas9‐MH* vector. To generate the *NLSS3* and *SHM1* overexpression constructs, the full‐length coding sequences (CDS) of *NLSS3* and *SHM1* were amplified from NPB and inserted into the *pUbi::NLSS3‐GFP‐3×FLAG* and *pUbi::SHM1‐RFP‐3×HA* vectors, respectively. For the *pACTIN1::SHM1 nlss3* complementation line, the full‐length CDS of *SHM1* was cloned into the *pCAMBIA2300‐ACTIN1* vector. All constructs were introduced into rice calli via *Agrobacterium tumefaciens*‐mediated transformation. The primers used for vector construction are listed in Table S3.

### 5.7 Subcellular Localization of NLSS3

The full‐length cDNA of *NLSS3* was amplified from NPB and cloned into the *pUbi::NLSS3‐GFP‐3×FLAG* vector. The successfully constructed NLSS3‐GFP fusion expression vector was introduced into rice protoplasts via polyethylene glycol (PEG)‐mediated transformation. Green fluorescent protein (GFP) signals were observed using laser scanning confocal microscopy.

### 5.8 GUS Staining Assay

GUS staining was conducted using transgenic rice lines expressing the *ProNLSS3‐GUS* construct. Samples, including roots and leaves from seedlings, as well as leaf sheaths, stems, and panicles collected at the heading stage, were incubated in GUS staining solution for 16 h at 37°C. Following staining, the tissues were transferred to ethanol for destaining and preservation before observation.

### 5.9 In Situ Hybridization

A 3‐day‐old seedling was collected and processed by Wuhan Pinuofei Biological Co, Ltd. The probe of NLSS3 was amplified and labeled using the DIG RNA Labeling Kit (Roche, Basel, Switzerland).

### 5.10 RNA Extraction and Quantitative Real‐Time PCR

Total RNA was isolated from samples using the Total RNA Miniprep kit (Axygen, China), and first‐strand cDNA was subsequently synthesized following the manufacturer's instructions. Quantitative PCR (qPCR) analysis was conducted on a CFX96 Real‐Time System. Each reaction mixture contained SYBR Premix Ex Taq and gene‐specific primers. The *UBQ5* gene was used as an internal reference for normalization; primers used for real‐time PCR are listed in Table S4.

### 5.11 Yeast Two‐Hybrid Assay

For the yeast two‐hybrid (Y2H) assay, the full‐length coding sequence (CDS) of *NLSS3* was fused in‐frame to the GAL4 DNA‐binding domain (BD) in the *pGBKT7* bait vector, generating the *pGBKT7‐NLSS3* construct. Similarly, the full‐length CDS of *SHM1* was fused to the GAL4 activation domain (AD) in the *pGADT7* prey vector, yielding *pGADT7‐SHM1*. To delineate the interacting domain, the full‐length *NLSS3* CDS was divided into four distinct segments, each of which was individually cloned into the *pGBKT7* vector. Various combinations of the bait and prey plasmids were co‐transformed into the Y2H Gold yeast strain (Coolaber, China).

### 5.12 Bimolecular Fluorescence Complementation Assay

The CDS of *NLSS3* was cloned into the *pSAT1A‐nEYFP‐N1* vector, and the CDS of *SHM1* was cloned into the *pSAT1A‐cEYFP‐N1* vector. The combinations YN‐NLSS3 + YC and YN + YC‐SHM1 served as negative controls. The constructed YN‐NLSS3 and YC‐SHM1 were co‐transformed into rice protoplasts via PEG‐mediated transformation.

### 5.13 Co‐Immunoprecipitation Assay

The full‐length coding sequences of *NLSS3* and *SHM1* were cloned into the *pCAMBIA1300‐35S::3×FLAG* and *pCAMBIA1300‐35S::GFP* vectors, respectively. The constructed *pCAMBIA1300‐35S::NLSS3‐3×FLAG* plasmid was transiently co‐expressed in rice protoplasts with either the empty *pCAMBIA1300‐35S::GFP* vector (negative control) or the *pCAMBIA1300‐35S::SHM1‐GFP* plasmid. The co‐immunoprecipitation assay was performed following a previously described method [59].

### 5.14 Pull‐Down Assay

The coding sequences of *NLSS3*, *NLSS3^A132P^*, and *SHM1* were cloned into the *pMal‐C5X* and *pGEX‐6p* vectors, respectively. The resulting MBP‐NLSS3, MBP‐NLSS3^A132P^, and GST‐SHM1 fusion constructs were expressed in *Escherichia coli* BL21 (Rosetta). Recombinant proteins were purified using an MBP‐tagged protein purification kit (Labled, China) and a GST‐tagged protein purification kit (Beyotime, China), following the manufacturer's protocol. The pull‐down assay was performed as described previously [8].

### 5.15 Split‐Luciferase Complementation Assay

For the split‐luciferase (Split‐LUC) assay, the coding sequences of *SHM1*, *NLSS3*, or *NLSS3^A132P^* were fused to the N‑terminal (nLUC) and C‑terminal (cLUC) fragments of firefly luciferase, respectively. The resulting constructs (SHM1‑nLUC, NLSS3‑cLUC, and NLSS3^A132P^‑cLUC) were expressed in *Agrobacterium*. Cultures carrying SHM1‑nLUC paired with either NLSS3‑cLUC or NLSS3^A132P^‑cLUC were mixed at a 1:1 ratio and co‑infiltrated into *N. benthamiana* leaves.

### 5.16 Cell‐Free Degradation Assay

A cell‐free degradation assay was performed using purified recombinant GST‐SHM1 protein and total protein extracts from 3‐week‐old seedlings of NPB and *nlss3‐cas* plants following a previously established method [60]. The degradation reaction was terminated at designated time points. Protein abundance was analyzed by immunoblotting using a GST‐specific antibody, with an Actin antibody serving as a loading control.

### 5.17 Semi‐In Vivo Ubiquitination Assay

The semi‐in vivo ubiquitination assay was conducted as previously described [18]. Purified GST‐SHM1 protein bound to glutathione‐sepharose beads was incubated with equal amounts of total protein extracts from NPB and *nlss3‐cas* seedlings. After the reaction, proteins were solubilized in SDS‐PAGE loading buffer and separated by SDS‐PAGE. Immunoblotting was performed using antibodies against ACTIN, Ubiquitin (Ub), and GST, with ACTIN serving as a loading control to verify consistent total protein input.

### 5.18 Autophagy Induction and Observation Assays

Autophagy was induced and inhibited as previously described, with modifications. For the induction of autophagy in protoplasts for immunoblotting, purified protoplasts expressing GFP‐SHM1 were treated with either the autophagy inducer rapamycin (10 µM) or the inhibitor chloroquine (100 µm) for 16 h [61, 62]. For the observation of autophagy induction in protoplasts via microscopy, protoplasts were treated with the inducer rapamycin (100 µM) together with concanamycin A (100 µM) for 30 min [63]. After treatment, protoplasts were collected for confocal microscopy to monitor the formation of GFP‐labeled puncta or for protein extraction to assess protein abundance.

For autophagy induction in whole plants for immunoblotting, two‐week‐old seedlings were treated with E64d (20 µm) or rapamycin (100 µM) for 4 h [64]. After treatment, samples were harvested for protein extraction and immunoblot analysis.

### 5.19 Quantification of Photorespiration Intermediates and Enzymes Activity

The contents of amino acids and enzymes activity of SHMT, HPR, and GDC in plant tissues were quantified using an enzyme‐linked immunosorbent assay (ELISA). The procedure was performed according to the manufacturer instruction (Meimian, China). Briefly, 0.1 g of plant tissue was ground in liquid nitrogen and homogenized in 1 mL of ice‐cold phosphate‐buffered saline (PBS) to extract total protein. Subsequently, the sample, biotinylated detection antibody, streptavidin‐HRP, and TMB substrate were sequentially added, followed by incubation at 37°C. The absorbance was measured at 450 nm. A standard curve was generated using a series of standard concentrations, and the corresponding glycine, serine, and SHMT levels were calculated accordingly.

For other intermediates of photorespiration chains, seedling samples were collected and milled. Relative content metabolites in rice samples were determined via UPLC‐MS/MS (Wuhan Metware Biotechnology Co., Ltd; http://www.metware.cn/).

### 5.20 Luciferase Activity Assay in Rice Protoplasts

To investigate the effect of natural variation in the *NLSS3* promoter region on its transcriptional activity, a transient expression assay was conducted in protoplasts. The promoter fragments of *NLSS3* were amplified from three representative cultivars, NPB (Hap1), 9311 (Hap2), and Zhefu 802 (Hap3). All amplified fragments were cloned into the *pGreenII 0800‐LUC* vector. The resulting recombinant plasmids were transfected into rice protoplasts. Luciferase activity was measured using a dual‐luciferase reporter assay system (Promega, USA) according to the manufacturer's instructions [8].

### 5.21 Statistical Analysis

All measured data were statistically analyzed using R Studio (4.1.1). The results are presented as the mean ± SD. Differences between groups were assessed using Student's *t*‐test or one‐way ANOVA with Tukey's test.

## Funding

This work was funded by Zhejiang Provincial Natural Science Foundation (Grant No. LD24C130001), National Natural Science Foundation of China (Grant No. 32188102, W2412006 and 32372125), Hainan Provincial Natural Science Foundation (Grant No. GHYF2025029, YBXM2527), the National Modern Agricultural Industry Technology System Project (Grant No. CARS‐01‐018), Special Support Program of Chinese Academy of Agricultural Sciences (Grant No. NKYCLJ‐C‐2021‐015 and CAAS‐ZDRW202401), the Fund of the Innovation Platform For Academicians of Hainan Province (YSPTZX202502).

## Conflicts of Interest

The authors declare no conflicts of interest.

## Supporting information




**Supporting file**: advs75700‐sup‐0001‐SuppMat.docx

## Data Availability

The data that support the findings of this study are available from the corresponding author upon reasonable request.

## References

[1] T. Sasaki and B. Burr , “International Rice Genome Sequencing Project: the Effort to Completely Sequence the Rice Genome,” Current Opinion in Plant Biology 3 (2000): 138–142, 10.1016/s1369-5266(99)00047-3. 10712951

[2] W. Guo , L. Chen , L. Herrera‐Estrella , D. Cao , and L. P. Tran , “Altering Plant Architecture to Improve Performance and Resistance,” Trends in Plant Science 25 (2020): 1154–1170, 10.1016/j.tplants.2020.05.009. 32595089

[3] H. Zhao , X. Liu , J. Wang , Q. Qian , and G. Zhang , “The Coordinated Regulation Mechanism of Rice Plant Architecture and Its Tolerance to Stress,” Frontiers in Plant Science 13 (2022): 1087378, 10.3389/fpls.2022.1087378. 36600918 PMC9807110

[4] A. Sasaki , M. Ashikari , M. Ueguchi‐Tanaka , et al., “A Mutant Gibberellin‐Synthesis Gene in Rice,” Nature 416 (2002): 701–702, 10.1038/416701a. 11961544

[5] T. Kuroha , K. Nagai , R. Gamuyao , et al., “Ethylene‐Gibberellin Signaling Underlies Adaptation of Rice to Periodic Flooding,” Science 361 (2018): 181–186, 10.1126/science.aat1577. 30002253

[6] S.‐Q. Guo , Y.‐X. Chen , Y.‐L. Ju , et al., “Fine‐Tuning Gibberellin Improves Rice Alkali–Thermal Tolerance and Yield,” Nature 639 (2025): 162–171, 10.1038/s41586-024-08486-7. 39880957

[7] Y. Jiao , Y. Wang , D. Xue , et al., “Regulation of OsSPL14 by OsmiR156 Defines Ideal Plant Architecture in Rice,” Nature Genetics 42 (2010): 541–544, 10.1038/ng.591. 20495565

[8] Y. Wang , Y. Lv , Y. Wen , et al., “GS2 Cooperates With IPA1 to Control Panicle Architecture,” New Phytologist 245 (2025): 2726–2743, 10.1111/nph.20412. 39887382 PMC11840411

[9] K. Miura , M. Ikeda , A. Matsubara , et al., “OsSPL14 Promotes Panicle Branching and Higher Grain Productivity in Rice,” Nature Genetics 42 (2010): 545–549, 10.1038/ng.592. 20495564

[10] J. Wang , L. Zhou , H. Shi , et al., “A Single Transcription Factor Promotes Both Yield and Immunity in Rice,” Science 361 (2018): 1026–1028, 10.1126/science.aat7675. 30190406

[11] F. Chen , H. Zhang , H. Li , et al., “IPA1 Improves Drought Tolerance by Activating SNAC1 in Rice,” BMC Plant Biology 23 (2023): 55, 10.1186/s12870-023-04062-9. 36698063 PMC9875436

[12] M. Jia , X. Meng , X. Song , et al., “Chilling‐Induced Phosphorylation of IPA1 by OsSAPK6 Activates Chilling Tolerance Responses in Rice,” Cell Discovery 8 (2022): 71, 10.1038/s41421-022-00413-2. 35882853 PMC9325753

[13] M. Jia , N. Luo , X. Meng , et al., “OsMPK4 Promotes Phosphorylation and Degradation of IPA1 in Response to Salt Stress to Confer Salt Tolerance in Rice,” Journal of Genetics and Genomics 49 (2022): 766–775, 10.1016/j.jgg.2022.06.009. 35803541

[14] X. Song , X. Meng , H. Guo , et al., “Targeting a Gene Regulatory Element Enhances Rice Grain Yield by Decoupling Panicle Number and Size,” Nature Biotechnology 40 (2022): 1403–1411, 10.1038/s41587-022-01281-7. 35449414

[15] M. Liu , Z. Shi , X. Zhang , et al., “Inducible Overexpression of Ideal Plant Architecture1 Improves Both Yield and Disease Resistance in Rice,” Nature Plants 5 (2019): 389–400, 10.1038/s41477-019-0383-2. 30886331

[16] G. Zhang , X. Hou , L. Wang , et al., “Photo‐Sensitive Leaf Rolling 1 Encodes a Polygalacturonase That Modifies Cell Wall Structure and Drought Tolerance in Rice,” New Phytologist 229 (2021): 890–901, 10.1111/nph.16899. 32858770

[17] J. Wang , J. Xu , L. Wang , et al., “Semi‐Rolled Leaf 10 Stabilizes Catalase Isozyme B to Regulate Leaf Morphology and Thermotolerance in Rice (Oryza sativa L.),” Plant Biotechnology Journal 21 (2023): 819–838, 10.1111/pbi.13999. 36597711 PMC10037157

[18] J. You , W. Xiao , Y. Zhou , et al., “The APC/CTAD1‐Wide Leaf 1‐Narrow Leaf 1 Pathway Controls Leaf Width in Rice,” The Plant Cell 34 (2022): 4313–4328, 10.1093/plcell/koac232. 35904763 PMC9614488

[19] M.‐L. Han , Q.‐Y. Lv , J. Zhang , et al., “Decreasing Nitrogen Assimilation Under Drought Stress by Suppressing DST‐Mediated Activation of Nitrate Reductase 1.2 in Rice,” Molecular Plant 15 (2022): 167–178, 10.1016/j.molp.2021.09.005. 34530166

[20] B. Saeed , C. Brillada , and M. Trujillo , “Dissecting the Plant Exocyst,” Current Opinion in Plant Biology 52 (2019): 69–76, 10.1016/j.pbi.2019.08.004. 31509792

[21] J. Liu , X. Zuo , P. Yue , and W. Guo , “Phosphatidylinositol 4,5‐Bisphosphate Mediates the Targeting of the Exocyst to the Plasma Membrane for Exocytosis in Mammalian Cells,” Molecular Biology of the Cell 18 (2007): 4483–4492, 10.1091/mbc.e07-05-0461. 17761530 PMC2043555

[22] B. He , F. Xi , X. Zhang , J. Zhang , and W. Guo , “Exo70 Interacts With Phospholipids and Mediates the Targeting of the Exocyst to the Plasma Membrane,” The EMBO Journal 26 (2007): 4053–4065, 10.1038/sj.emboj.7601834. 17717527 PMC2230670

[23] Y. Zhang , C. M. Liu , A. M. Emons , and T. Ketelaar , “The Plant Exocyst,” Journal of Integrative Plant Biology 52 (2010): 138–146, 10.1111/j.1744-7909.2010.00929.x. 20377676

[24] F. Cvrčková , M. Grunt , R. Bezvoda , et al., “Evolution of the Land Plant Exocyst Complexes,” Frontiers in Plant Science 3 (2012): 159, 10.3389/fpls.2012.00159. 22826714 PMC3399122

[25] T. Pecenková , V. Markovic , P. Sabol , I. Kulich , and V. Žárský , “Exocyst and Autophagy‐Related Membrane Trafficking in Plants,” Journal of Experimental Botany 69 (2017): 47–57, 10.1093/jxb/erx363. 29069430

[26] I. Kulich , T. Pecenková , J. Sekeres , et al., “Arabidopsis Exocyst Subcomplex Containing Subunit EXO70B1 Is Involved in Autophagy‐Related Transport to the Vacuole,” Traffic 14 (2013): 1155–1165, 10.1111/tra.12101. 23944713

[27] A. K. Acheampong , C. Shanks , C.‐Y. Cheng , G. E. Schaller , Y. Dagdas , and J. J. Kieber , “EXO70D Isoforms Mediate Selective Autophagic Degradation of Type‐A ARR Proteins to Regulate Cytokinin Sensitivity,” Proceedings of the National Academy of Sciences 117 (2020): 27034–27043, 10.1073/pnas.2013161117. PMC7604425 33051300

[28] J. C. De la Concepcion , K. Fujisaki , A. R. Bentham , et al., “A Blast Fungus Zinc‐Finger Fold Effector Binds to a Hydrophobic Pocket in Host Exo70 Proteins to Modulate Immune Recognition in Rice,” Proceedings of the National Academy of Sciences 119 (2022): 2210559119, 10.1073/pnas.2210559119. PMC9618136 36252011

[29] H. Hou , J. Fang , J. Liang , et al., “OsExo70B1 Positively Regulates Disease Resistance to Magnaporthe Oryzae in Rice,” International Journal of Molecular Sciences 21 (2020): 7049, 10.3390/ijms21197049. 32992695 PMC7582735

[30] D. Wu , J. Guo , Q. Zhang , et al., “Necessity of Rice Resistance to Planthoppers for OsEXO70H3 Regulating SAMSL Excretion and Lignin Deposition in Cell Walls,” New Phytologist 234 (2022): 1031–1046, 10.1111/nph.18012. 35119102 PMC9306520

[31] Y. Xing , N. Wang , T. Zhang , et al., “SHORT‐ROOT 1 Is Critical to Cell Division and Tracheary Element Development in Rice Roots,” The Plant Journal 105 (2021): 1179–1191, 10.1111/tpj.15095. 33231904

[32] S. Rosa‐Téllez , A. Alcántara‐Enguídanos , F. Martínez‐Seidel , et al., “The Serine–Glycine–One‐Carbon Metabolic Network Orchestrates Changes in Nitrogen and Sulfur Metabolism and Shapes Plant Development,” The Plant Cell 36 (2024): 404–426, 10.1093/plcell/koad256. 37804096 PMC10827325

[33] R. Waditee‐Sirisattha , D. Sittipol , Y. Tanaka , and T. Takabe , “Overexpression of Serine Hydroxymethyltransferase From Halotolerant Cyanobacterium in Escherichia Coli Results in Increased Accumulation of Choline Precursors and Enhanced Salinity Tolerance,” FEMS Microbiology Letters 333 (2012): 46–53, 10.1111/j.1574-6968.2012.02597.x. 22587350

[34] J. I. Moreno , R. Martín , and C. Castresana , “Arabidopsis SHMT1, A Serine Hydroxymethyltransferase That Functions in the Photorespiratory Pathway Influences Resistance to Biotic and Abiotic Stress,” The Plant Journal 41 (2005): 451–463, 10.1111/j.1365-313X.2004.02311.x. 15659103

[35] W. Jiang , Z. Wang , Y. Li , et al., “FERONIA Regulates Salt Tolerance in Arabidopsis by Controlling Photorespiratory Flux,” The Plant Cell 36 (2024): 4732–4751, 10.1093/plcell/koae246. 39197037 PMC11530776

[36] Y. Liu , C. Mauve , M. Lamothe‐Sibold , et al., “Photorespiratory Serine Hydroxymethyltransferase 1 Activity Impacts Abiotic Stress Tolerance and Stomatal Closure,” Plant, Cell & Environment 42 (2019): 2567–2583, 10.1111/pce.13595. 31134633

[37] S. Fu , K. Wang , T. Ma , et al., “An Evolutionarily Conserved C4HC3‐type E3 Ligase Regulates Plant Broad‐Spectrum Resistance against Pathogens,” The Plant Cell 34 (2022): 1822–1843, 10.1093/plcell/koac055. 35171277 PMC9048923

[38] D. Wang , H. Liu , S. Li , G. Zhai , J. Shao , and Y. Tao , “Characterization and Molecular Cloning of a Serine Hydroxymethyltransferase 1 (OsSHM1) in Rice,” Journal of Integrative Plant Biology 57 (2015): 745–756, 10.1111/jipb.12336. 25641188

[39] C. Fang , P. Zhang , L. Li , et al., “Serine Hydroxymethyltransferase Localised in the Endoplasmic Reticulum Plays a Role in Scavenging H_2_O_2_ to Enhance Rice Chilling Tolerance,” BMC Plant Biology 20 (2020): 236, 10.1186/s12870-020-02446-9. 32456700 PMC7249644

[40] J. K. Zhu , “Plant Salt Tolerance,” Trends in Plant Science 6 (2001): 66–71, 10.1016/s1360-1385(00)01838-0. 11173290

[41] M. G. Mostofa , M. M. Rahman , T. K. Ghosh , et al., “Potassium in Plant Physiological Adaptation to Abiotic Stresses,” Plant Physiology and Biochemistry 186 (2022): 279–289, 10.1016/j.plaphy.2022.07.011. 35932652

[42] R. H. Köhler , W. R. Zipfel , W. W. Webb , and M. R. Hanson , “The Green Fluorescent Protein as a Marker to Visualize Plant Mitochondria In Vivo,” Plant Journal 11 (1997): 613–621, 10.1046/j.1365-313x.1997.11030613.x. 9107047

[43] Z. Liu , Q. Yang , P. Wu , et al., “Dynamic Monitoring of TGW6 by Selective Autophagy During Grain Development in Rice,” New Phytologist 240 (2023): 2419–2435, 10.1111/nph.19271. 37743547

[44] R. Liu , R. Zhang , Y. Yang , X. Liu , and Q. Gong , “Monitoring Autophagy in Rice With GFP‐ATG8 Marker Lines,” Frontiers in Plant Science 13 (2022): 866367, 10.3389/fpls.2022.866367. 35548298 PMC9083259

[45] Y. Z. Pe , “A Review on Modeling of Responses of Photosynthesis to Light and CO_2_ ,” Chinese Journal of Plant Ecology 34 (2010): 727–740, 10.3773/j.issn.1005-264x.2010.06.012.

[46] S. Holden , M. Bergum , P. Green , et al., “A Lineage‐Specific Exo70 Is Required for Receptor Kinase–Mediated Immunity in Barley,” Science Advances 8 (2022): abn7258, 10.1126/sciadv.abn7258. PMC9258809 35857460

[47] K. Fujisaki , Y. Abe , A. Ito , et al., “Rice Exo70 Interacts With a Fungal Effector, AVR ‐Pii, and is Required for AVR ‐Pii‐Triggered Immunity,” The Plant Journal 83 (2015): 875–887, 10.1111/tpj.12934. 26186703

[48] B. Tu , L. Hu , W. Chen , et al., “Disruption of OsEXO70A1 Causes Irregular Vascular Bundles and Perturbs Mineral Nutrient Assimilation in Rice,” Scientific Reports 5 (2015): 18609, 10.1038/srep18609. 26691393 PMC4686888

[49] L. Synek , N. Schlager , M. Elias , M. Quentin , M.‐T. Hauser , and V. Zarský , “AtEXO70A1, a Member of a Family of Putative Exocyst Subunits Specifically Expanded in Land Plants, Is Important for Polar Growth and Plant Development,” The Plant Journal 48 (2006): 54–72, 10.1111/j.1365-313X.2006.02854.x. 16942608 PMC2865999

[50] V. Žárský , “Exocyst Functions in Plants: Secretion and Autophagy,” Febs Letters 596 (2022): 2324–2334, 10.1002/1873-3468.14430. 35729750

[51] L. Wang , P. Hou , W. Ma , et al., “Unveiling EXOC4/SEC8: A Key Player in Enhancing Antiviral Immunity by Inhibiting the FBXL19‐STING1‐SQSTM1 Signaling Axis,” Autophagy 21 (2025): 2578–2596, 10.1080/15548627.2025.2511077. 40413753 PMC12758185

[52] M. Jia , X. Liu , H. Xue , et al., “Noncanonical ATG8–ABS3 Interaction Controls Senescence in Plants,” Nature Plants 5 (2019): 212–224, 10.1038/s41477-018-0348-x. 30664732 PMC6368864

[53] H. Zhou , J. Zhao , Y. Yang , et al., “UBIQUITIN‐SPECIFIC PROTEASE16 Modulates Salt Tolerance in Arabidopsis by Regulating Na^+^/H^+^ Antiport Activity and Serine Hydroxymethyltransferase Stability,” The Plant Cell 24 (2012): 5106–5122, 10.1105/tpc.112.106393. 23232097 PMC3556978

[54] S. C. Kalhan and R. W. Hanson , “Resurgence of Serine: an Often Neglected but Indispensable Amino Acid,” Journal of Biological Chemistry 287 (2012): 19786–19791, 10.1074/jbc.R112.357194. 22566694 PMC3370164

[55] R. Douce , J. Bourguignon , M. Neuburger , and F. Rébeillé , “The Glycine Decarboxylase System: A Fascinating Complex,” Trends in Plant Science 6 (2001): 167–176, 10.1016/s1360-1385(01)01892-1. 11286922

[56] L. M. Voll , A. Jamai , P. Renne , H. Voll , C. R. McClung , and A. P. M. Weber , “The Photorespiratory Arabidopsis shm1 Mutant is Deficient in SHM1,” Plant Physiology 140 (2006): 59–66, 10.1104/pp.105.071399. 16339799 PMC1326031

[57] G.‐H. Zhang , S.‐Y. Li , L. Wang , et al., “LSCHL4 From Japonica Cultivar, Which is Allelic to NAL1, Increases Yield of Indica Super Rice 93‐11,” Molecular Plant 7 (2014): 1350–1364, 10.1093/mp/ssu055. 24795339 PMC4115278

[58] J. Xu , L. Wang , Y.‐X. Wang , et al., “Reduction of OsFLW7 Expression Enhanced Leaf Area and Grain Production in Rice,” Science Bulletin 62 (2017): 1631–1633, 10.1016/j.scib.2017.11.013. 36659380

[59] Y. Wang , Y. Hou , J. Qiu , et al., “Abscisic Acid Promotes Jasmonic Acid Biosynthesis via a ‘SAPK10‐bZIP72‐ AOC ’ Pathway to Synergistically Inhibit Seed Germination in Rice (Oryza sativa),” New Phytologist 228 (2020): 1336–1353, 10.1111/nph.16774. 32583457 PMC7689938

[60] S. Chang , Q. Yang , W. Chu , et al., “Lysine Deacetylase TaSRT1 Mediates Wheat Drought Tolerance by Deacetylating TaDT‐A to Reduce its Protein Stability and Transcriptional Activity,” Plant Biotechnology Journal 23 (2025): 1650–1667, 10.1111/pbi.14613. 39977256 PMC12018820

[61] J. He , H. Peng , M. Wang , et al., “1‐Induced Fibrogenesis Through Activating Autophagy via PI3K/AKT/mTOR Pathway in MRC‐5 Cells,” Acta Biochimica et Biophysica Sinica 52 (2020): 810–820, 10.1093/abbs/gmaa067. 32638014

[62] J. Guo , H. Wang , W. Guan , et al., “A Tripartite Rheostat Controls Self‐Regulated Host Plant Resistance to Insects,” Nature 618 (2023): 799–807, 10.1038/s41586-023-06197-z. 37316670 PMC10284691

[63] S. Zhang , L. Jiang , H. Chen , et al., “Gibberellin Triggers ATG8‐Dependent Autophagic Degradation of DELLA Proteins to Promote Seed Germination and Skotomorphogenesis Under Nutrient Starvation in Arabidopsis,” Molecular Plant 18 (2025): 2101–2118, 10.1016/j.molp.2025.10.011. 41108080

[64] M. Jia , X. Liu , H. Xue , et al., “Noncanonical ATG8–ABS3 Interaction Controls Senescence in Plants,” Nature Plants 5 (2019): 212–224, 10.1038/s41477-018-0348-x. 30664732 PMC6368864

