# Supplementary material for: NLSS3 Impairs SHM1 Autophagic Degradation to Regulate Leaf Morphology and Salt Tolerance in Rice
Source: Adv Sci (Weinh). 2026 Jun 3;13(45):e75700. doi: 10.1002/advs.75700 (PMC13336080; doi:10.1002/advs.75700)
Supplement: Supplementary file 1 [file ADVS-13-e75700-s001.docx]

Supporting Information

**NLSS3 Impairs SHM1 Autophagic Degradation to Regulate Leaf Morphology and Salt Tolerance in Rice**

*Xiong Liu^1†^, Yulu Yang**^1†^, Zhiqi Hao^1†^, Jing Xu^1,3^, Huibo Zhao^1,2^, Qiang Zhang^1,2^, Deyong Ren^1^, Xia Li^1,2^, Guojun Dong^1^, Lan Shen^1^, Li Zhu^1,2^, Jiang Hu^1,2^, Zhenyu Gao^1^, Qing Li^1^,* *Qian Qian^1,2,4*^, and* *Guangheng Zhang^1,2*^*

**Figure S1.** Comparison of stem traits between NPB and *nlss3.*

a) Hand sections from the 1st to 4th internodes of NPB and *nlss3* plants at heading stage. Scale bar, 1 cm. b) Culm diameter of NPB and *nlss3*. Data represent means ± SD (*n* = 5). c) Cross-sections of the 1st internode of NPB and *nlss3*, with red and blue arrows indicating small and large vascular bundles, respectively. Scale bars, 200 μm. d) Number of vascular bundles of 1st internode in NPB and *nlss3.* Data represent means ± SD (*n* = 5). The significance of all the above data was determined by Student's *t*-test. ** for *P* < 0.01.

**Figure** **S2.** Phenotypic differences between NPB and *nlss3* under salt stress.

a) Representative images of NPB and *nlss*3 before and after salt stress at seedling stage. Scale bars, 10 cm. b-c) Biomass of NPB and *nlss3* before and after stress. Shoot dry weight (b), root dry weight (c). Data represent means ± SD (*n* = 5). d-f) Activities of ROS-scavenging enzymes in NPB and *nlss3* under salt stress at seedling stage. POD activity (d), SOD activity (e), CAT activity (f). Data represent means ± SD (*n* = 4). The significance of all the above data was determined by Student's *t*-test. ** for *P* < 0.01; * for *P* < 0.05; ns, not significant.

**Figure S3.** Relative expression level of major K^+^ uptake genes in NPB and *nlss3.*

a-d) Relative expression level of major K^+^ uptake genes in NPB and *nlss3* at seedling stage*. HAK8* (a), *HAK1* (b), *HAK16* (c), *AKT1* (d)*.* Data represent means ± SD (*n* = 4). The significance of all the above data was determined by Student's *t*-test. ** for *P* < 0.01; * for *P* < 0.05; ns, not significant.

**Figure S4.** Photosynthetic parameters in NPB and *nlss3* at heading stage.

a) Photosynthetic rate of NPB and *nlss*3. Data represent means ± SD (*n* = 5). b) Intercellular CO_2_ concentration of NPB and *nlss*3. Data represent means ± SD (*n* = 5). c) Stomatal conductance of NPB and *nlss*3. Data represent means ± SD (*n* = 5). d) Transpiration rate of NPB and *nlss*3. Data represent means ± SD (*n* = 5). The significance of all the above data was determined by Student's *t*-test. ** for *P* < 0.01; * for *P* < 0.05; ns, not significant.

**Figure S5.** Yield-related traits of NPB and *nlss3.*

a) Spike morphology of NPB and *nlss3*. Scale bars, 5 cm. b) Grain size and scanning electron microscopy (SEM) images of the outer and inner epidermis of the glumes of NPB and *nlss3*. Grain size (upper), outer epidermis of the glume (lower left), inner epidermis of the glume (lower right). Scale bars, 1 cm, 200 μm, 100 μm. c-h) Comparison of yield-traits between NPB and *nlss3*. Panicle length (c), primary branch numbers (d), second branch numbers (e), grain numbers per panicle (f), yield per plant (g), thousand-grain weight (h). Data represent means ± SD (*n* = 10). i–j) Comparison of grain size between NPB and *nlss3*, grain length (i), grain width (j). Data represent means ± SD (*n* = 10). k–l) Cell width of the outer and inner epidermis of the glumes in NPB and *nlss3*. Inner glume (k), outer glume (l). Data represent means ± SD (*n* = 10). The significance of all the above data was determined by Student's *t*-test. ** for *P* < 0.01; * for *P* < 0.05; ns, not significant.

**Figure S6.** Map-based cloning of *NLSS3*.

**Figure S7.** Functional characterization of *NLSS3*.

a) Cross-sections of flag leaf in NPB, *NLSS3* knockout and overexpression lines at tillering stage. Scale bars, 200 μm. b) Mutant type of *NLSS3* knockout lines. c) Number of large veins of NPB, *NLSS3* knockout and overexpression lines. Data represent means ± SD (*n* = 5). Significance was determined by one-way ANOVA with Tukey's test. Significant differences between groups are marked with different letters. d) Number of small veins of NPB, *NLSS3* knockout and overexpression lines. Data represent means ± SD (*n* = 5). Significance was determined by one-way ANOVA with Tukey's test. Significant differences between groups are marked with different letters.

**Figure S8.** GUS staining and in situ hybridization of *NLSS3* in different tissues.

a) GUS staining of *NLSS3* in different tissues. Scale bars, 0.5 cm. b) GUS staining of *NLSS3* in leaf primordia. Scale bar, 50 μm. VB indicates vascular bundle. c) In situ hybridization of *NLSS3* in leaf primordia. Left was the negative control, Right was the magnification of the box in middle. Scale bars, left and middle 100 μm, right 50 μm. VB indicates vascular bundle. d) Relative expression level of *NLSS3* in small, large veins and leaf at tillering stage. Data represent means ± SD (*n* = 5).

**Figure S9.** The A132P mutation in NLSS3 weakens its interaction with SHM1.

**Figure S10.** NLSS3 attenuates SHM1 autophagic degradation.

a) In vitro degradation assay of GST-SHM1 in NPB and *nlss3-cas* plants under CQ treatment. b) Protein abundance fitting curve of the in vitro degradation assay. Relative data were log_2_-transformed c) Comparison of SHM1 protein abundance in NPB and *NLSS3-OE* plants under normal and salt conditions.

**Figure S11.** Content of photorespiratory intermediates and enzymes in NPB and *nlss3*.

a) Heatmap of relative metabolite abundance of photorespiratory intermediates in NPB and *nlss3* at seedling stage. Relative metabolite abundance data were log_2_-transformed. Colors represent the transformed values, with red indicating high abundance and blue indicating low abundance. 2-PG: 2-phosphoglycolate, 2-OG: 2-oxoglutarate, Ser: serine, Gly: glycine. b) HPR (hydroxypyruvate reductase) activity of NPB and *nlss3* at seedling stage. Data represent means ± SD (*n* = 5). c) GDC (glycine decarboxylase complex) activity of NPB and *nlss3* at seedling stage. Data represent means ± SD (*n* = 5). Significance was determined by Student's *t*-test. ns, not significant.

**Figure S12.** Yield-related traits of *SHM1* overexpression lines.

a) Representative images of whole plants of NPB and *SHM1* overexpression plants at heading stage. Scale bars, 10 cm. b-c) Plant height and effective panicle numbers of NPB and *SHM1* overexpression plants, plant height (b), effective panicle numbers (c). Data represent means ± SD (*n* = 10). d-h) Panicle traits of NPB and *SHM1* overexpression plants, panicle length (d), primary branch numbers (e), second branch numbers (f) and seed setting rate (g). Data represent means ± SD (*n* = 10). The significance of all the above data was determined by Student's *t*-test, ** for *P* < 0.01; * for *P* < 0.05; ns, not significant.

**Figure S13.** SHM1 protein levels in different plants.

a) SHM1 protein levels in NPB, *nlss3-cas*, and *SHM1-OE/nlss3-cas* plants. b) SHM1 protein levels in NPB, *nlss3*, and *NLSS3-com* plants.

**Figure S14.** SHM1 activity and protein level in 9311 (Hap2) and 9311-NIL (Hap1) plants carrying different haplotype of *NLSS3.*

a) SHM1 activity in 9311 (Hap2) and 9311-NIL (Hap1) plants at seedling stage. Data represent means ± SD (*n* = 4). b) SHM1 protein level 9311 (Hap2) and 9311-NIL (Hap1) plants at seedling stage. Significance of all the above data was determined by Student's *t*-test.; * for *P* < 0.05; ns, not significant.

**Figure S15.** Yield-related traits of the 9311 (Hap2) and 9311-NIL (Hap1) carrying different haplotype of *NLSS3*

a) Representative images of panicle in 9311 (Hap2) and 9311-NIL(Hap1) plants. Scale bars, 10 cm. b) Effective panicle numbers of 9311 (Hap2) and 9311-NIL (Hap1) plants. Data represent means ± SD (*n* = 10). c) Panicle length of 9311 (Hap2) and 9311-NIL (Hap1) plants. Data are presented as mean ± SD (*n* = 10). d) Primary branch numbers of 9311 (Hap2) and 9311-NIL (Hap1) plants. Data represent means ± SD (*n* = 5). e) Second branch numbers of 9311 (Hap2) and 9311-NIL (Hap1) plants. Data represent means ± SD (*n* = 10). f) Grain numbers per panicle of 9311 (Hap2) and 9311-NIL (Hap1) plants. Data represent means ± SD (*n* = 10). g) Seed setting rate of 9311 (Hap2) and 9311-NIL (Hap1) plants. Data represent means ± SD (*n* = 10). h) Yield per plant of 9311 (Hap2) and 9311-NIL (Hap1) plants. Data represent means ± SD (*n* = 5) The significance of all the above data was determined by Student's *t*-test. ** for *P* < 0.01; * for *P* < 0.05; ns, not significant.

**Figure S16.** Autophagosomes in NPB and *nlss3* root cells

a) Autophagosomes in NPB and *nlss3* root cells at seedling stage. Red arrows point out the autophagosomes. Scale bar, 1 μm. b) Number of autophagosomes per cell in NPB and *nlss3* root cells. Data represent means ± SD (*n* = 5). Significance of all the above data was determined by Student's *t*-test. ns, not significant.

**Table S1.** Genetic analysis of F_2_ population of hybrid combination.

| Hybrid combination | Number of plants in F_2_ population | | | χ^2^ |
| --- | --- | --- | --- | --- |
|  | Narrow leaves | Normal leaves | Total |  |
| *nlss3/*TN1 | 456 | 1478 | 1934 | 3.98 |
| 93-11/*nlss3* | 344 | 940 | 245 | 4.36 |

**Table S2.** Primers used for map-based cloning.

| Primer name | | Forward primer (5’-3’) | Reverse primer (5’-3’) |
| --- | --- | --- | --- |
| **B3-11 | CACCGTTTGGTTTGGCAG | | AGCATGGTGTACCTCGTCG |
| **B3-12 | TGTTGTTTTGGAGATTTGAAGG | | GAGGCGAAAAGTCACGTAAGC |
| **B3-14 | AGGATTTGGGCTGTCAATGC | | AGCCTACCACAAGACATATAATCG |
| **B3-15 | TCCAGTCCACTAAAAGTTTTC | | ATTATGCTATAGGCTTTGAGC |
| *N-1 | CATGAGCTTAACCATGTTCT | | ATTTGATGTTCAACTCAGGG |
| *N-2 | GTGAAACGTTGGATGATTTT | | TAAAAGCCGTTGGAAATAGA |
| *N-3 | GGAGCTCGGATGAATTAGAT | | GCCCTTGAGTCTATGACAAG |
| *N-4 | CAGCGACGAGCAGTACAC | | AGGTCACTAGTTATTCAACA |
| *N-5 | CTGTAATACAGTCAACGAATAAG | | ATTGGCTCCGCTTATGGGCTA |
| *N-6 | ACCTTCTCTCTCTCTCCTGG | | GGATTTGGAAGGAGACGG |
| *N-7 | AGAAGTGAAACAAGCTCCAA | | TCAACCTGATCCTGAAAGTT |
| *N-8 | ATCCTTCGTTGACATAGCTG | | AAAAATGATTGGAATGGAAC |
| *N-9 | TAGAGCGGTTAAAATGGAAG | | ATATTTGTAATGTGGCGGAT |

Note: **for primary mapping; *for fine mapping

**Table S3.** Primers used for vector construction.

| Primer name | Forward primer (5’-3’) | Reverse primer (5’-3’) |
| --- | --- | --- |
| NLSS3-COM | aattcgagctcggtaccACCGCTATTGTCGTCATTGTC | ctagaggatccccgggtaccCCAATAACTCCCAAAGCTCAAC |
| NLSS3-GFP | TGTTACTTCTGCAGGAGCTCATGGCGCTCGTCGCCGTGGC | CTCACCATGGATCCGGTACCCTGTACATGGGCCAATTTGTCC |
| NLSS3-OE | TGTTACTTCTGCAGGAGCTCATGGCGCTCGTCGCCGTGGC | CTCACCATGGATCCGGTACCTCACTGTACATGGGCCAATTTGTCC |
| BD-NLSS3(AA 24-102) | CATGGAGGCCGAATTCGCAGCGCTCATCGACAAGTG | GGATCCCCGGGAATTCGGTCGACAGGAGAAGCTCCA |
| BD-NLSS3(AA 24-187) | CATGGAGGCCGAATTCGCAGCGCTCATCGACAAGTG | GGATCCCCGGGAATTCTGACTGCACCTTGCCGTCCA |
| BD-NLSS3(AA 188-448) | CATGGAGGCCGAATTCTGGCACACCGCCGCGGGATT | GGATCCCCGGGAATTCCACCAGCACGTCTCGCCACG |
| BD-NLSS3(AA 449-556) | CATGGAGGCCGAATTCGTTAAGGAAGCGGTGGCGAC | GGATCCCCGGGAATTCTCACTGTACATGGGCCAATTTGTCC |
| BD-NLSS3 (AA 1-556) | CATGGAGGCCGAATTCATGGCGCTCGTCGCCGTGGC | GGATCCCCGGGAATTCTCACTGTACATGGGCCAATTTGTCC |
| AD-SHM1 | CCATGGAGGCCAGTGAATTCATGGCCATGGCGACGGCGCT | TGCCCACCCGGGTGGAATTCTTAGTTCTTGTACTTCATGG |
| YN-NLSS3 | gaggactccggactcAGATCTATGGCGCTCGTCGCCGTGGC | cgaagcttgagctcgAGATCTTCACTGTACATGGGCCAATTTGTCC |
| YC-SHM1 | TACAAGTCCGGACTCAGATCTATGGCCATGGCGACGGCGCT | CGAAGCTTGAGCTCGAGATCTTTAGTTCTTGTACTTCATGG |
| NLSS3-Flag | TGGAGAGGACAATTGGGTACCATGGCGCTCGTCGCCGTGGC | GGTATCGATGGATCCGTCGACCTGTACATGGGCCAATTTGT |
| SHM1-GFP | CGCTCTAGAACTAGTGGATCCATGGCCATGGCGACGGCGCT | GATAAGCTTGATATCGAATTCGTTCTTGTACTTCATGGTTT |
| SHM1-RFP | CTTCTGCAGGAGCTCGatggccatggcgacggcgct | GCTCGCTCATGGATCCgttcttgtacttcatggttt |
| NLSS3-U3 | GGCAGTGCCTTGTTCGCGCGCAG | AAACCTGCGCGCGAACAAGGCAC |
| SHM1-U3 | GGCAGCGGAACCATGTAGTAGAGC | aaacGCTCTACTACATGGTTCCGC |
| MBP-NLSS3 | GACGGATCCGAATTCATGGCGCTCGTCGCCGTGGC | ACCTGCAGGGAATTCTCACTGTACATGGGCCAATTTGTCC |
| GST-SHM1 | cccctgggatccccggaattcATGGCCATGGCGACGGCGCT | ctcgagtcgacccgggaattcTTAGTTCTTGTACTTCATGG |
| 2300-SHM1 | ggggatcctctagagtcgacATGGCCATGGCGACGGCG | agccctggcatgcctgcaggTTAGTTCTTGTACTTCATGG |
| SHM1-OE  0800-NLSS3 | CTTCTGCAGGAGCTCGatggccatggcgacggcgct  agggcgaattgggtacCGGATTCACCACCATCGTCG | GCTCGCTCATGGATCCTTAGTTCTTGTACTTCATGG  ggcggccgctctagaaGATGGATGGACCTTCAATTC |
| NLSS3-cLUC | cgtcccggggcggtacccggATGGCGCTCGTCGCCGTGGC | acgaacgaaagctctgcaggTCACTGTACATGGGCCAATTTGTCC |
| SHM1-nLUC  GUS-NLSS3 | gggacgagctcggtacccggATGGCCATGGCGACGGCGCT  AGCTCGGTACCCGGGGATCCCGGATTCACCACCATCGTCG | gcgtacgagatctggtcgacGTTCTTGTACTTCATGG  ctcagatctaccatGGATCCGATGGATGGACCTTCAATTC |

**Table S4.** Primers used for qRT-PCR.

| **Primer name** | **Forward primer (5’-3’)** | **Reverse primer (5’-3’)** |
| --- | --- | --- |
| OsUBQ5-RT | AACCAGCTGAGGCCCAAGA | ACGATTGATTTAACCAGTCCATGA |
| NLSS3-RT | GGCGCGTGGTATTCTGATGA | TCATTACGTAGCGGGTGAGC |
| SHM1-RT | ACGGCTACCAGACTGATACT | CTTGTCACAAACCTTCCGCA |
| HAK1-RT | GTTGATGATGCTGATGTTGGAAG | CCAACACTTTCAGCTGAAAC |
| HAK8-RT | GGGACTCATGGAAGACAACC | GAACACGAAAGAGAGCACAC |
| HAK16-RT | CATGCCAACAATCAGTAAG | CATTTGCAAGTAAGCAAACC |
| AKT1-RT | AGAGATCCTTGATTCACTGCC | TCCGAAACCAGTTGAAAAAT |
